# Long-term outcome of sinusoidal obstruction syndrome secondary to hematopoietic stem cell transplantation

**DOI:** 10.1016/j.jhepr.2026.101986

**Published:** 2026-08-06

**Authors:** Edilmar Alvarado-Tapias, Alexandre Sayadi, Lise Bertin, Aurélie Plessier, François Durand, Andreu Ferrero-Gregori, Valérie Paradis, David Michonneau, Pierre-Emmanuel Rautou

**Affiliations:** 1 Gastroenterology and Hepatology Department, Hospital de la Santa Creu i Sant Pau, Department of Medicine, Universitat Autònoma de Barcelona, Barcelona, Spain; 2 Group of Inflammatory Diseases, Institut de Recerca Sant Pau (IR-SANT PAU), Barcelona, Spain; 3 Center for Biomedical Research in Liver and Digestive Diseases Network (CIBERehd), Instituto de Salud Carlos III, Madrid, Spain; 4 AP-HP, Hôpital Beaujon, Service de Pathologie, DMU DREAM, Clichy, France; 5 Université Paris-Cité, Inserm, Centre de recherche sur l'inflammation, UMR 1149, Paris, France; 6 AP-HP, Hôpital Beaujon, Service d'Hépatologie, DMU DIGEST, Centre de Référence des Maladies Vasculaires du Foie, FILFOIE, ERN RARE-LIVER, Clichy, Paris, France; 7 Hematology and transplantation unit, Saint-Louis Hospital, AP-HP, Paris, France; 8 INSERM U976, human immunology, pathophysiology, immunotherapy, IHU Institut de la Leucémie Paris Saint-Louis, Paris-Cité University, Paris, France

**Keywords:** Veno-occlusive disease, Liver, Portal hypertension, Porto-sinusoidal vascular liver disorder, Idiopathic portal hypertension, Endothelium

## Abstract

**Background & Aims:**

Sinusoidal obstruction syndrome (SOS) is a potentially severe complication of conditioning regimens in hematopoietic stem cell transplantation (HSCT). Its long-term outcome is unknown. The aim of this study was to investigate the long-term liver outcome of patients with SOS.

**Methods:**

We retrospectively analyzed the outcome of all patients with histologically proven SOS related to HSCT conditioning who survived 6 months or more after HSCT. Twenty-two patients with SOS were included.

**Results:**

Median follow-up after SOS was 6.1 years. During this period, 10 patients (45%) developed signs of portal hypertension and eight (36%) of them had liver-related complications (ascites, n = 8; spontaneous bacterial peritonitis, n = 1; hepatic encephalopathy, n = 3; variceal bleeding, n = 3). Cumulative incidence of signs of portal hypertension was 27% at 3 years. Patients who developed signs of portal hypertension were less likely to have received defibrotide (hazard ratio: 0.096; *p* <0.001) and had lower mean arterial pressure than those who did not. Of the 10 patients who developed signs of portal hypertension during follow-up, three underwent a second liver biopsy; all biopsies showed features of porto-sinusoidal vascular disorder. Cumulative incidence of death, considering non-liver-related death as a competing event, was significantly higher in patients who developed signs of portal hypertension than in those who did not (hazard ratio: 10.7 [1.27–89.9]).

**Conclusions:**

Development of signs of portal hypertension and liver-related complications are common in the long-term after SOS following HSCT, especially when patients did not receive defibrotide, and this affects patients’ survival. Features consistent with a porto-sinusoidal vascular disorder may be seen in liver histology in a subset of patients at the long-term follow-up after SOS.

**Impact and implications:**

Long-term liver outcome of SOS following conditioning regimens in HSCT is unknown. This study demonstrates that signs of portal hypertension and liver-related complications are common during long-term follow-up after SOS following HSCT. Treatment of SOS with defibrotide seems to be associated with a lower risk of developing portal hypertension. Liver histology at the long-term follow-up after SOS may show features consistent with porto-sinusoidal vascular disorder. SOS following HSCT might thus be removed from the porto-sinusoidal vascular disorder exclusion criteria.

## Highlights

• Histological findings suggest that SOS may progress to porto-sinusoidal vascular disorder (PSVD) in some patients.
• Absence of defibrotide treatment at SOS diagnosis is independently associated with later portal hypertension.
• Ascites, hepatic encephalopathy, and variceal bleeding are common and associated with reduced long-term survival.
• Long-term hepatic surveillance is warranted after SOS following HSCT, including liver tests, elastography, and imaging.

## Graphical abstract

**Figure:**
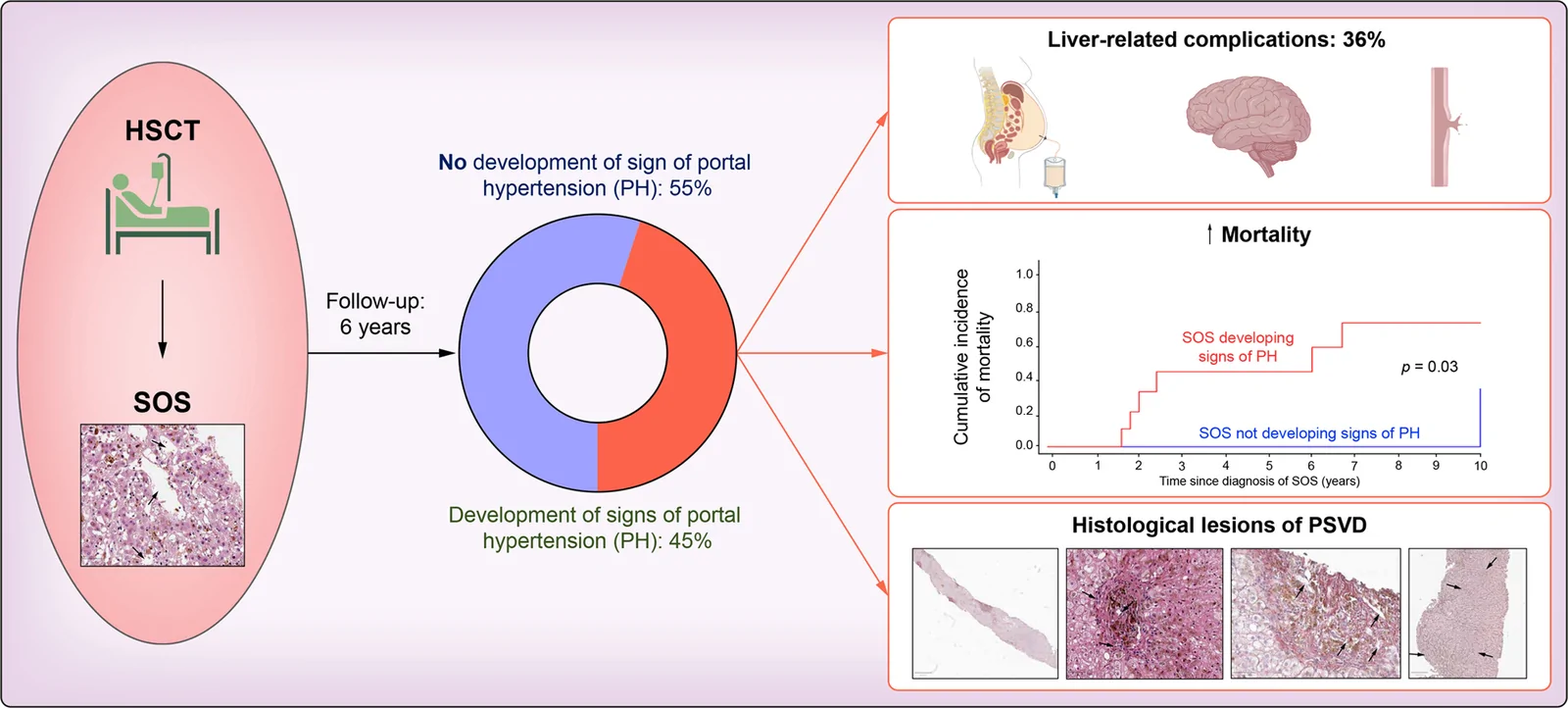

## Introduction

Sinusoidal obstruction syndrome (SOS), formerly known as hepatic veno-occlusive disease (VOD), is a potentially lethal form of liver injury characterized by toxic/inflammatory damage to liver sinusoidal endothelial cells.^1^^,^^2^ These endothelial cells then undergo necrosis and extrusion into the sinusoids, resulting in partial or complete occlusion of small hepatic venules.^1^^,^^2^ SOS occurs most commonly as a complication of myeloablative chemotherapy regimens (high-dose with or without total-body irradiation) used before hematopoietic stem cell transplantation (HSCT), but may also occur following exposure to platinum-based chemotherapy or pyrrolizidine alkaloids.^2^

In the setting of HSCT, clinical manifestations of SOS include right upper quadrant pain, hepatomegaly, weight gain attributable to edema, ascites, transfusion-refractory thrombocytopenia, and jaundice. Mild SOS typically resolves progressively over a few weeks, with 70% of patients recovering spontaneously, under mainly supportive treatment. By contrast, severe cases of SOS have a very high mortality rate (over 80%).^1^ Once SOS is resolved, the patients’ outcomes are unknown, so it is currently not possible to inform patients and physicians about the liver prognosis after SOS or to adapt medical follow-up.

Therefore, this study aimed to investigate the long-term hepatic outcome of patients with liver biopsy-proven SOS.

## Patients and methods

### Patients

We retrospectively analyzed a prospective database including all patients who underwent a transjugular liver biopsy and a hepatic venous pressure gradient (HVPG) measurement at Hôpital Beaujon, Clichy, France, between 1994 and 2023, to identify all patients referred from the Hematology department of Saint-Louis Hospital for clinical suspicion of SOS in the setting of HSCT.

We included in this study all patients fulfilling all the following criteria: (i) a clinical suspicion of SOS according to the 2023 criteria of the European Society for Blood and Marrow Transplantation, based on two of the following features:^1^ serum bilirubin ≥2 mg/dl (*i.e.* 34 μmol/L); painful hepatomegaly; weight gain >5%; ascites; ultrasound and/or elastography findings suggestive of SOS; (ii) histological lesions of SOS at liver biopsy; and (iii) a survival of ≥6 months or more after HSCT conditioning. The decision to perform a liver biopsy was based on the treating physician’s judgment.

Exclusion criteria were a known history of chronic liver disease before HSCT conditioning, recurrence of hematological disease during the first 6 months after HSCT, or loss to follow-up after HSCT.

This study was conducted in accordance with the ethical guidelines of the 1975 Declaration of Helsinki and was approved by the institutional review board (CPP Sud Méditerranée V, Nice, France – Ethics approval number 2021-A02148-33). This observational, retrospective study of a prospective patient database was designed, conducted, and written in accordance with the STROBE guidelines.

### Liver and spleen stiffness measurements

Liver stiffness measurement (LSM) and spleen stiffness measurement (SSM) were measured during follow-up using vibration-controlled transient elastography (VCTE) (FibroScan®, Echosens, Paris, France) or 2D shear wave elastography (Aixplorer® SuperSonic Imagine, Aix-en-Provence, France) by trained operators. Measurements were obtained after at least 2 h fasting. For VCTE, 10 valid measurements with an IQR/median ratio <30% and a success rate >60% were required for reliability. For 2D shear wave elastography, we considered an IQR/median ratio for LSM or SSM <30% as reliable, as suggested by the Society of Radiologists in Ultrasound.^3^

LSM values <10.0 kPa in patients with signs of portal hypertension were considered as strongly suggestive of porto-sinusoidal vascular disorder (PSVD).^4^ SSM values ≥40 kPa were considered strongly suggestive of portal hypertension.^5^

### Hemodynamic study

Hepatic and right-heart catheterizations were performed using a technique previously described.^6^ After overnight fasting, local anesthesia was performed, and an introducer was placed under ultrasound guidance using the Seldinger technique. A catheter was then placed in the right or median hepatic vein. Between 1994 and 2012, wedge hepatic venous pressure was measured by ‘wedging’ a tip-curved catheter (Cook, HNB7.0-38-100-P–NS–MPA, Bloomington, Indiana, in the United States) into a small branch of a hepatic vein. Adequate occlusion was confirmed by injection of 5 ml of iodinated radiological contrast medium. A measurement was considered valid when two consecutive measurements from two different veins differed by <1 mmHg. Since 2013, wedge hepatic venous pressure has been measured by inflating a 7 French balloon catheter (Lemaitre Vascular; Edwards Lifesciences™, Irvine, California, United States) in the right or median hepatic vein. Adequate occlusion was confirmed by injection of 5 ml of iodinated radiological contrast medium. Then, free hepatic venous pressure was obtained. HVPG was calculated as the difference between wedged hepatic venous pressure and free hepatic venous pressure. A measurement was considered valid when two consecutive measurements differed by <1 mmHg. Permanent tracings were recorded using the Mac-Lab recording system (GE Healthcare, Milwaukee, Wisconsin, United States). Right-heart hemodynamic measurements, including pulmonary artery pressure, right atrial pressure, and pulmonary capillary wedge pressure, were also performed using a Swan-Ganz catheter (Edwards Life Sciences™, Irvine, United States). The thermodilution method was used to measure the cardiac index, with an average of three to five consecutive measurements obtained by injecting 10 ml of cold (1–4 °C) saline.^6^

### Histological analysis

#### Biopsies performed at the diagnosis of SOS and during follow-up

Liver tissue samples obtained from patients were formalin-fixed and paraffin-embedded. Hematein-Eosin-Safran, Picrosirius Hematoxylin, and Perls-stained tissue sections were analyzed. Reticulin stain was systematically performed when sufficient material was available (*i.e.* for all but one biopsy). All liver biopsies performed at the diagnosis of SOS and during follow-up were retrospectively reviewed by two pathologists, including an expert liver pathologist (VP) and a fellow pathologist (AS) with experience in histological assessment of vascular liver diseases. Both were unaware of the patients’ clinical data.

Histopathological evaluation of the biopsies performed at the diagnosis of SOS confirmed the initial diagnosis of SOS in all but one case, which was thus excluded from the present study. As we included early- and late-onset SOS, SOS was defined as (a) dilation and engorgement of sinusoids with red blood cells, associated with various perivenular and/or central vein changes, including necrosis of perivenular hepatocytes, sinusoidal fibrosis as well as subendothelial edema and hemorrhage in the venule wall; and/or (b) fibrous obstruction of central vein associated or not with other lesions. For systematic assessment of histological lesions of SOS, the following features were collected based on previous works:^7^ parenchymal lesions (sinusoidal dilatation, perisinusoidal hemorrhage, peliosis, hepatocellular damage, nodularity); venular lesions (central vein and portal vein lesions, *i.e.* endothelial cell rounding); and vascular fibrosis (sinusoidal fibrosis, portal vein fibrosis, fibrous obstruction of central veins). For sinusoidal dilatation, a semi-quantitative scoring was used: 0, no lesion; 1, sinusoidal dilatation limited to centrilobular sinusoids; 2, extensive sinusoidal dilatation affecting centrilobular areas up to mediolobular sinusoids; 3, sinusoidal dilatations extending to periportal zones.^7^ Perisinusoidal hemorrhage, peliosis, and hepatocellular damage were assessed as present or absent. Venular lesions and vascular fibrosis were assessed as follows: 0, absent; 1, <50% of veins/portal tracts/sinusoids; and 2, ≥50% of veins/portal tracts/sinusoids throughout the whole biopsy. Overall, SOS severity was determined based on the extent of histological lesions associated with SOS: mild (centrolobular), moderate (centro- and mediolobular), or severe (panlobular).

Histological features associated with PSVD were also evaluated on all biopsies (diagnosis of SOS and follow-up). Portal vein stenosis was evaluated as follows: 0, absent; 1, <50% of portal veins; and 2, ≥50% of portal veins throughout the entire biopsy. Portal tract abnormalities (*i.e.* hypervascularized portal tracts, herniated portal vein branches, and periportal abnormal vessels) were assessed as present or absent. Nodular regeneration was assessed according to Wanless *et al.*,^8^ ranging from 0 (absent) to 3 (nodular regenerative hyperplasia [NRH]).^8^ Sinusoidal dilatation and sinusoidal fibrosis were assessed as described above. Diagnosis of PSVD was made according to the 2019 VALDIG criteria and endorsed by Baveno VII,^8^^,^^9^ except for the minimal liver biopsy size, which was set at 15 mm or more, according to recently published data.^9^ The 2026 PSVD or non-cirrhotic portal fibrosis diagnostic score was also calculated.^10^

Other lesions assessed in all biopsies included portal fibrosis (according to METAVIR staging), bile deposits, and steatosis. For steatosis, a semi-quantitative assessment of the proportion of involved hepatocytes was used as follows: 0, absent; 1, mild (5–33%); 2, moderate (33–66%); and 3, severe (>66%). Steatohepatitis was assessed as present or absent. Iron deposits were not assessed, as they were present in virtually all patients in the context of HSCT and a history of transfusion.

For patients with sufficient remaining material, immunostaining for keratin 7 (K7) and CD34 was performed to assess ischemic hepatocyte changes and sinusoidal capillarization.^11^

### Follow-up

Patients' outcomes were retrieved from their medical records until June 2025, including liver blood tests, blood cell count, and liver images.

During follow-up, the development of signs of portal hypertension was assessed in all patients using platelet count and abdominal imaging, and in some patients using SSM and/or upper gastrointestinal tract endoscopy. HVPG was not routinely performed during follow-up.

Signs of portal hypertension were classified according to the criteria proposed by VALDIG, and endorsed by Baveno VII:^12^^,^^13^ (i) specific signs of portal hypertension included varices (gastric, esophageal, or ectopic), portal hypertensive bleeding, and portosystemic collaterals at imaging; (ii) non-specific signs of portal hypertension included a history of ascites, platelet count <150×10^9^/L, and spleen size ≥13 cm in the largest axis. The same criteria were used for calculating the 2026 PSVD or non-cirrhotic portal fibrosis diagnostic score, except that small varices were classified as a non-specific sign of portal hypertension, and SSM >40 kPa as a specific sign.

Liver-related complications were defined as the development of ascites, hepatic encephalopathy (grade II or higher according to West-Haven), or portal hypertensive-related bleeding. Spontaneous bacterial peritonitis and portal vein thrombosis were also recorded.

### Statistical analysis

Quantitative variables were expressed as median (IQR) and categorical variables as frequencies (%). Comparisons of independent quantitative and qualitative variables between groups were performed using the Mann–Whitney *U* test and Χ^2^ or Fisher’s exact tests, as appropriate. For time-to-event analyses, the start of follow-up was the initiation of the conditioning regimen for HSCT. Cumulative incidence was calculated by considering the number of new cases of the condition of interest during a defined period, divided by the population at risk at baseline. In this study, we utilized competing risk analysis to account for the presence of mutually exclusive events that may influence the primary outcome (liver-related complications), considering non-liver-related death as a competing risk event. Specifically, we calculated the cumulative incidence function to estimate the probability of each competing event over time, providing a more accurate representation of event-specific risks. To compare the cumulative incidence curves between groups, Gray's test was used, as it allows for a robust assessment of differences in the presence of competing risks. To determine predictors of signs of portal hypertension (specific or not) after HSCT during follow-up, the Fine–Gray subdistribution hazards model was used.^14^ Initially, variables with a *p* value <0.10 in univariate analyses were selected as candidates for inclusion in the model. Subsequently, a backward elimination process was applied, whereby variables were iteratively removed based on their statistical significance until the final model included only those variables that maintained a significant association with the subdistribution hazard of the outcome of interest. Statistical analyses were performed using the SPSS statistical package 29.0 software (SPSS Inc., Chicago, IL, USA), and R software (R Foundation for Statistical Computing, Vienna, Austria).

## Results

### Characteristics of the patients

**Fig. 1:**
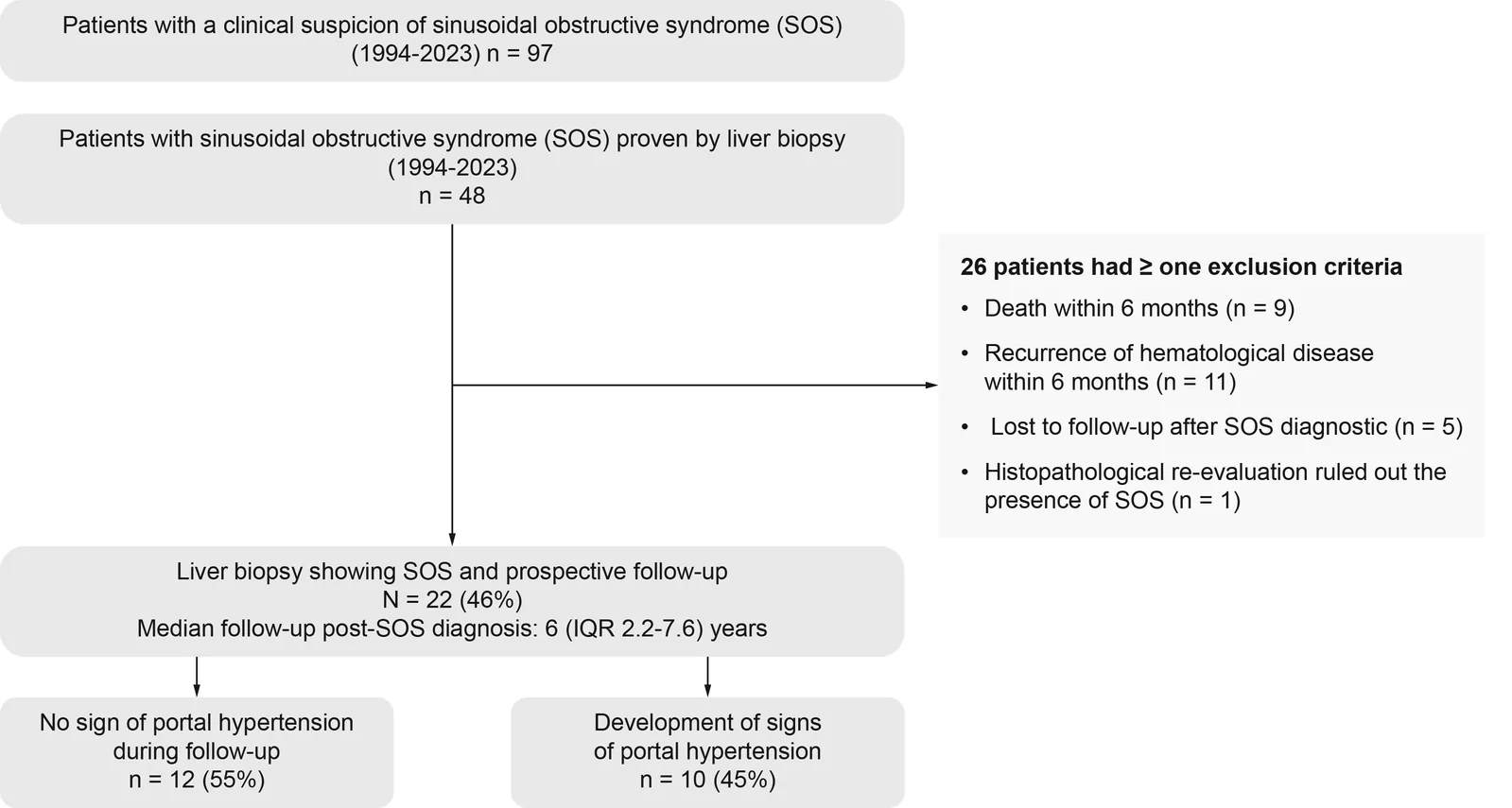
Flowchart of the study. FU, follow-up; PH, portal hypertension; SOS, sinusoidal obstructive syndrome.

**Fig. 2:**
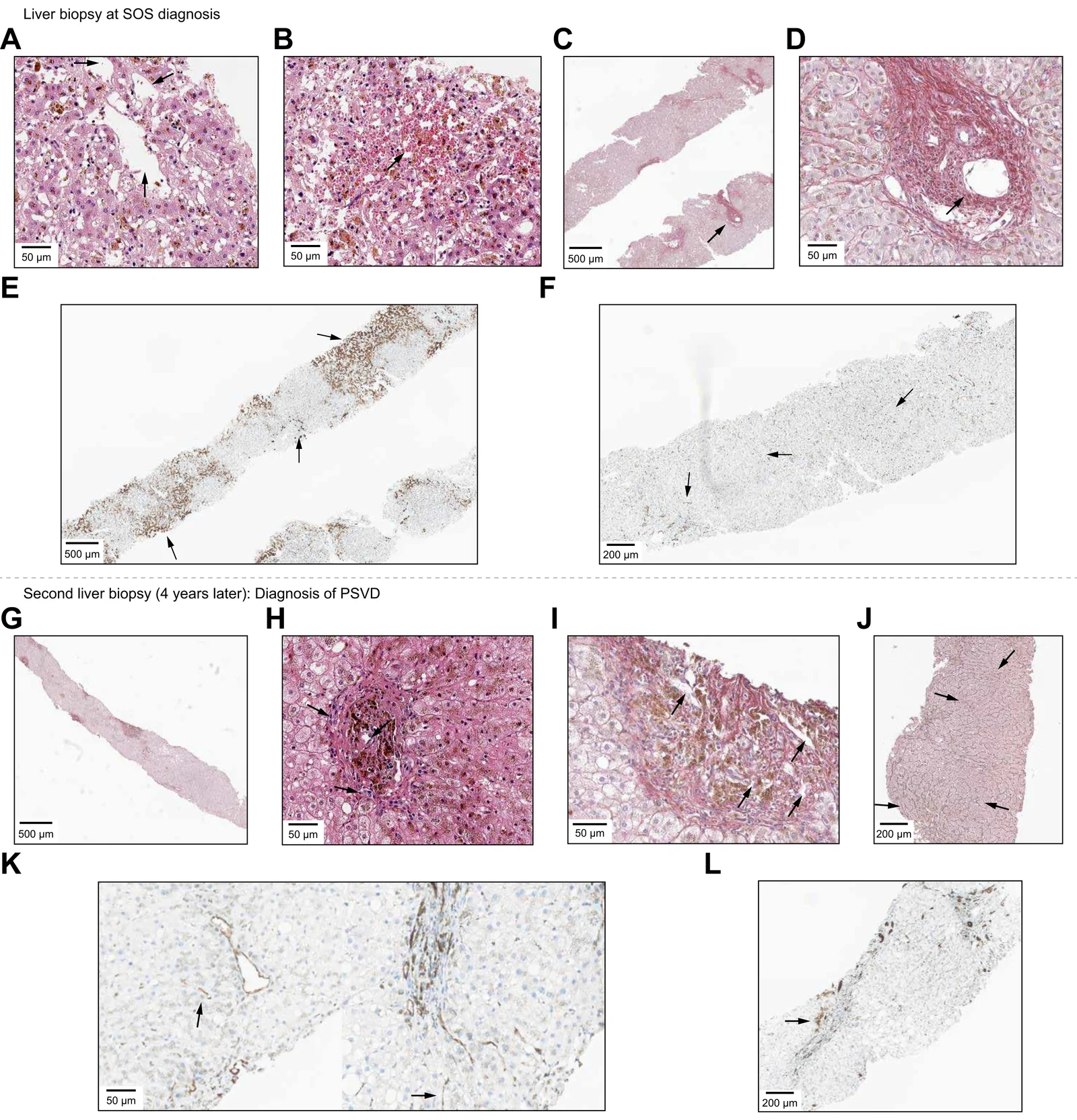
Patient #2. First liver biopsy: SOS diagnosis. (A) Hematoxylin–Eosin–Safran (HES) staining, 200×. Marked sinusoidal dilatation (arrows) surrounding the centrolobular vein (arrowhead). (B) HES staining, 200×. Sinusoidal dilatation is associated with sinusoidal congestion (defined by numerous erythrocytes in sinusoidal dilatation, arrow). Brown deposits in Kupffer cells and hepatocytes are iron deposits, stained by Perls stain (not shown). (C) Picro Sirius Hematoxylin (PSH) staining, 20×. Low magnification shows periportal fibrosis without septal fibrosis (arrowhead). Reticulin staining showed mild foci of regeneration, without complete nodular and regenerative hyperplasia (NRH, not shown). (D) PSH staining, 200×. All portal tracts had well-recognized portal veins (arrowhead). (E) Keratin 7 (K7) immunostaining, 20×. K7 highlights marked ischemic hepatocyte changes in zone 2 (mediolobular) and zone 3 (centrolobular) areas (arrows), without involvement of zone 1 (periportal, arrowhead). (F) CD34 immunostaining, 50×. Sinusoidal capillarization was heterogeneous, involving zone 1, 2 (arrows), and 3 (arrowhead). *Second liver biopsy (4 years later): Diagnosis of PSVD.* (G) PSH staining, 20×. Low magnification showed periportal fibrosis without any septal fibrosis nor cirrhosis. (H) HES staining, 200×. Portal vein stenosis was seen in numerous portal tracts, with easily recognizable artery (arrowhead), bile duct, and ductules (arrows) without portal vein. (I) PSH staining, 250×. Numerous small-sized vascular profiles (arrowheads) without any recognizable portal vein. (J) Reticulin stain, 50×. NRH with foci of regenerative hepatocyte plates (arrows) surrounded by atrophic ones (arrowheads). (K) CD34 immunostaining, 200×. CD34 highlighted mild sinusoidal capillarization in zone 3 (left, arrow) and zone 1 (right, arrow). (L) K7 immunostaining, 50×. Presence of a few periportal ischemic hepatocytes (arrow).CD, cluster differentiation; HES, Hematoxylin–Eosin–Safran; K7. Keratin 7; NRH. nodular regenerative hyperplasia; PSH, Picro Sirius Hematoxylin; PSVD, portal sinusoidal vascular disorder; SOS, sinusoidal obstructive syndrome.

During the study period, 48 patients were referred to our hepatic hemodynamic laboratory for a clinical suspicion of SOS. During the same period, SOS was diagnosed clinically in only 49 other patients who were not referred for liver biopsy. Of the 48 patients referred for biopsy, 26 had exclusion criteria, therefore 22 patients were included in the present study (Fig. 1). All of them had liver imaging before HSCT, showing no evidence for chronic liver disease. Their baseline demographic, hematological, clinical, laboratory, and hemodynamic characteristics at the time of liver biopsy for SOS diagnosis are shown in Table 1. None had HBV or HCV infection. Eleven of them were included in a previous study by our group.^15^ A detailed analysis of liver histological lesions is presented in Table S1, with examples shown in Fig. 2, and Figs. S1 and S2. SOS histological lesions were graded as mild (centrolobular), moderate (centro- and mediolobular), and severe (panlobular) in five, nine, and eight patients, respectively.^7^ Four patients also had histological lesions of PSVD, including NRH alone in one (Patient 4), portal vein stenosis in ≥50% of the portal tract alone in one (Patient 1), and both lesions in two patients (Patients 10 and 12). K7 immunostaining could be performed in eight patients and highlighted ischemic hepatocytes changes in five patients, in periportal (zone 1, n = 2) and/or centrolobular (zone 3, n = 4) areas. CD34 immunostaining was performed on eight patients and showed sinusoidal capillarization in all cases, with variable distribution across the lobule (Fig. 2 and Figs. S1 and S2). None of the patients had advanced liver fibrosis.

**Table 1:** Clinical, analytical, and hemodynamic characteristics at the time of liver biopsy of the 22 patients with histologically proven SOS and a follow-up of 6 months or more after HSCT conditioning.

	All patients with SOS (N = 22)		Patients not developing signs of portal hypertension during follow-up (n = 12)		Patients developing signs of portal hypertension during follow-up (n = 10)		*p* value
	n =	n (%) or median (IQR)	n =	n (%) or median (IQR)	n =	n (%) or median (IQR)	
Age (years)	22	46 (38–51)	12	46 (31–50)	10	46 (40–54)	0.565
Male sex (%)	22	17 (77)	12	9 (75)	10	8 (80)	1.000
Metabolic risk factors∗ (%)	22	10 (45)	12	5 (42)	10	5 (50)	1.000
History of alcohol use disorder (%)	22	4 (18)	12	1 (8)	10	3 (30)	0.293
Body mass index (kg/m^2^)	21	24 (21–27)	12	24 (22–25)	9	27 (21–30)	0.583

**Laboratory data before conditioning**							
Prothrombin index (%)	9	98 (93–99)	4	97 (95–98)	5	98 (86–99)	1.000
INR	9	161.00 (1.0–1.15)	9	1.00 (1.00–1.1)		71.00 (1.0–1.23)	0.413
Platelet count (×10^9^/L)	15	162 (55–184)	8	152 (34–222)	7	162 (78–177)	0.827
Serum AST (IU/L)	15	38 (31–42)	8	37 (30–40)	7	42 (34–44)	0.287
Serum ALT (IU/L)	15	40 (34–50)	8	40 (34.5–55)	7	36 (34–44)	0.580
Serum GGT (IU/L)	15	74 (44–141)	8	132 (58–152)	7	70 (38–82)	0.374
Serum total bilirubin (μmol/L)	15	8 (5–12)	8	10 (7–12)	7	6 (5–8)	0.436
Serum creatinine (μmol/L)	13	60 (55–70)	7	60 (57–66)	6	63 (52–75)	0.789
Serum albumin (g/L)	9	38 (37–39)	4	39 (37–41)	5	38 (37–39)	0.823

**Hematological disease history**							0.516
HL-NHL (%)	22	8 (36)	12	4 (33)	10	4 (40)	
Acute leukemia (%)	22	9 (41)	12	4 (33)	10	5 (50)	
Chronic leukemia (%)	22	3 (14)	12	2 (17)	10	1 (10)	
Others (%)	22	2 (9)	12	2 (17)	10	0 (0)	
Ursodeoxycholic acid (prophylaxis) (%)	22	12 (55)	12	9 (75)	10	3 (30)	0.084
AlloHSCT/AutoHSCT/no†(%)	22	8 (36)/10 (46)/4 (18)	12	5 (42)/5 (42)/2(17)	10	3 (30)/5 (50)/2 (20)	0.852
Time between conditioning or HSCT and signs of SOS (days)	22	19 (14–30)	12	21 (14–30)	10	19 (16–27)	0.898
Time between conditioning or HSCT and liver biopsy showing SOS (days)	22	32 (23–37)	12	31 (24–38)	10	32 (23–37)	0.955

**Features suggesting SOS**							
Total bilirubin ≥2 mg/dl (%)	22	9 (41)	12	6 (50)	10	3 (30)	0.415
Hepatomegaly (yes) (%)	21	21 (100)	12	12 (100)	9	9 (100)	1.000
Weight gain >5% (%)	22	12 (55)	12	5 (42)	10	7 (70)	0.231
Ascites (%)	22	16 (73)	12	8 (67)	10	8 (80)	0.646
Liver stiffness (kPa)	10	15 (14–17)	5	14 (8–14)	5	16 (16–17)	0.150
Liver stiffness >8 kPa (%)	10	10 (100)	5	3 (60)	5	5 (100)	0.444
Ultrasound features suggestive of SOS‡	22	22 (100)	10	10 (100)	12	12 (100)	1.000
Spleen size (larger axis) (cm)	19	12 (10–14)	10	11 (10–14)	9	14 (10–14)	0.808
Defibrotide treatment (%)	22	8 (36)	12	7 (58)	10	1 (10)	**0.031**
Severity grading of SOS (mild/moderate/severe/very severe)§	22	(3/5/12/2)	12	(2/3/6/1)	10	(1/2/6/1)	0.949

**Laboratory data at liver biopsy proving SOS**							
Prothrombin index (%)	22	79 (65–93)	12	70 (60–86)	10	86 (76–97)	0.086
INR	22	1.10 (1.02–1.24)	12	1.15 (1.05–1.3)	10	1.06 (1.01–1.19)	0.160
Platelet count (×10^9^/L)	21	45 (38–89)	12	48 (43–75)	9	43 (34–159)	0.837
Serum AST (IU/L)	22	60 (39–149)	12	90 (56–158)	10	49 (35–66)	0.074
Serum ALT (IU/L)	22	88 (52–197)	12	106 (73–198)	10	56 (45–114)	0.070
Serum GGT (IU/L)	22	149 (77–371)	12	158 (129–488)	**10**	126 (66–187)	0.193
Serum total bilirubin (μmol/L)	22	28 (11–45)	12	34 (15–50)	10	19 (10–30)	0.334
Serum creatinine (μmol/L)	22	71 (56–88)	12	71 (60–75)	10	67 (56–94)	0.824
Serum albumin (g/L)	19	31 (30–37)	12	32 (30–35)	7	31 (30–37)	0.903
HVPG (mmHg)	22	11 (7–14)	12	11 (9–14)	10	11 (6–14)	0.630
Right atrial pressure (mmHg)	21	4 (2–5)	11	3 (1–5)	10	4 (3–5)	0.406
Heart rate (bpm)	22	94 (75–108)	12	91 (71–108)	10	98 (82–105)	0.544
Mean arterial pressure (mmHg)	22	92 (80–108)	12	95 (90–114)	10	84 (69–100)	**0.053**

Data are presented as median (IQR) or n (%). Comparison of quantitative and qualitative variables was done using the Mann–Whitney *U* test and the Χ^2^ or Fisher’s exact tests, as appropriate. Bolded values indicate statistically significant differences (*p* <0.05).

AlloHSCT, allogenic hematopoietic stem cell transplantation; ALT, alanine aminotransferase level; AST, aspartate aminotransferase level; AutoHSCT, auto hematopoietic stem cell transplantation; Bpm, beats per minute; GGT, gamma-glutamyl transferase; HL, Hodgkin lymphoma; HSCT, hematopoietic stem cell transplantation; HVPG, hepatic venous pressure gradient; INR, international normalized ratio; NHL, non-Hodgkin lymphoma; SOS, sinusoidal obstruction syndrome.

∗ Metabolic risk factors included overweight (BMI ≥25 kg/m^2^), diabetes mellitus, arterial hypertension, and/or dyslipidemia.

† Some patients received conditioning before HSCT but did not ultimately undergo HSCT.

‡ Ultrasound in SOS following HSCT can detect non-specific abnormalities including: (i) hepatomegaly (>15 cm in the midclavicular line), (ii) splenomegaly (≥13 cm in the larger axis) (iii) gallbladder wall thickening, (iv) dilatation of the main portal vein (≥13 mm) (v) ascites, (vi) paraumbilical vein visualization, (vii) decrease mean velocity of the portal vein, (viii) hepatofugal flow or no flow in portal vein, and (ix) hepatic artery resistive index ≥0.75.

§ SOS clinical severity according to the 2023 EBMT criteria.

### Development of signs of portal hypertension during the follow-up

**Fig. 3:**
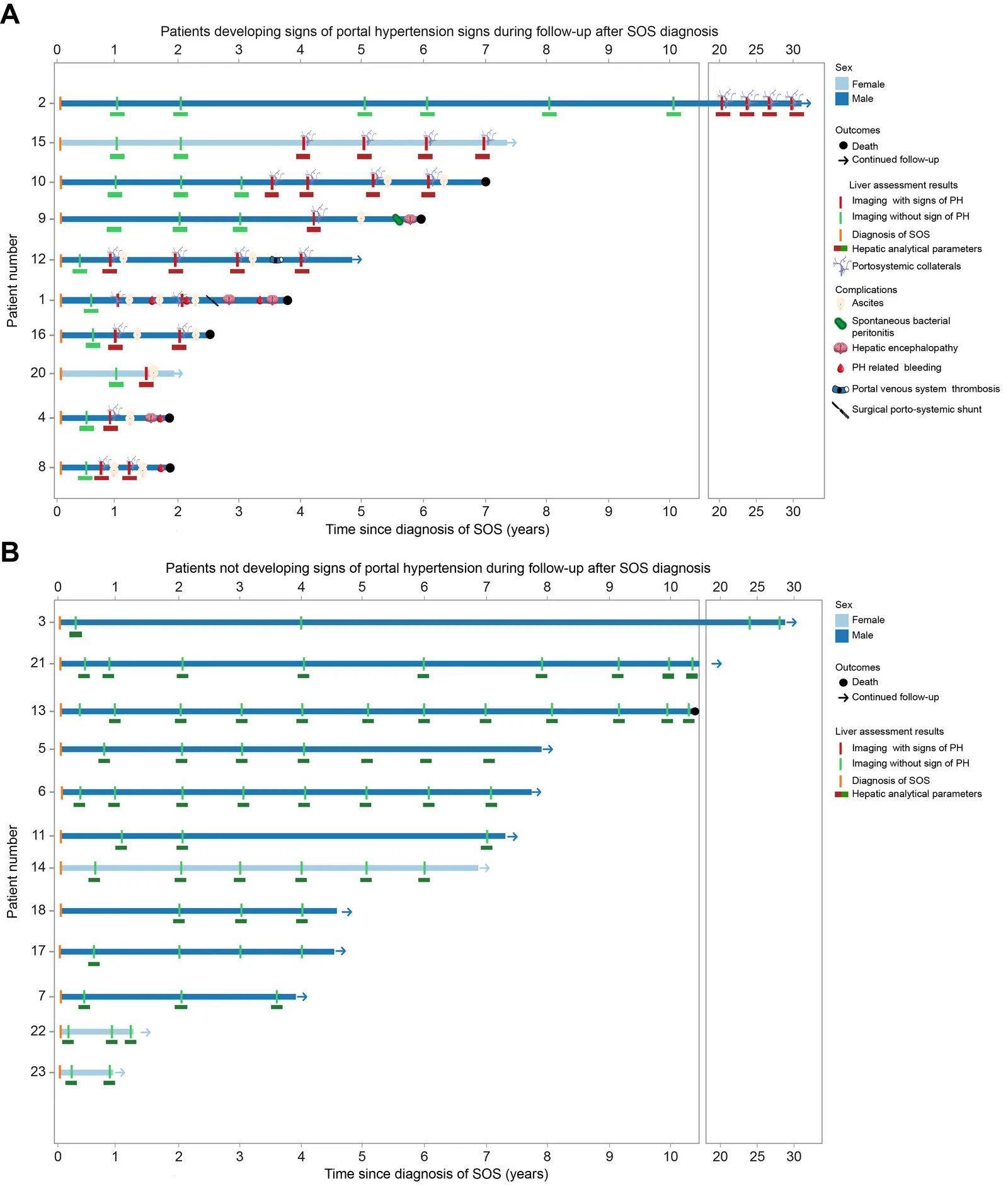
Clinical outcomes of patients with **(A)** and without **(B) the** appearance of signs of portal hypertension during follow-up after SOS diagnosis. PH, portal hypertension; SOS, sinusoidal obstructive syndrome.

**Fig. 4:**
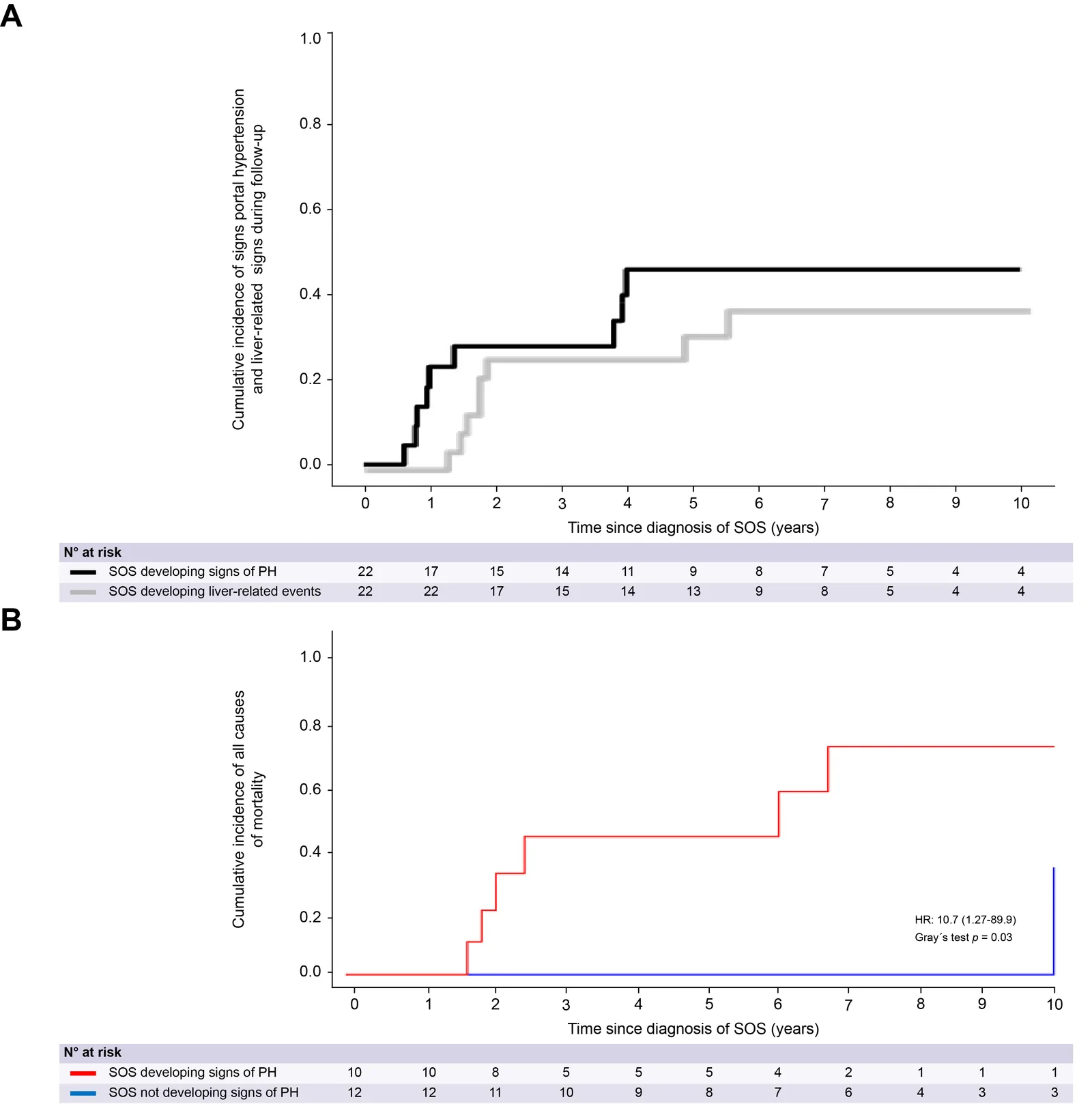
Cumulative incidence of portal hypertension and mortality in patients with SOS. (A) Cumulative incidence of patients with SOS developing signs of portal hypertension during follow-up (black line). Cumulative incidence of SOS patients developing liver-related complications during follow-up (gray line). (B) Cumulative incidence of mortality in SOS patients during follow-up, considering all the causes. PH, portal hypertension; SOS, sinusoidal obstructive syndrome.

Of the 22 patients included, 10 patients (45%) developed signs of portal hypertension, after a median follow-up of 1.3 years (IQR, 0.9–4.2) after SOS diagnosis, including splenomegaly (n = 4), thrombocytopenia (*<*150×10^9^) (n = 7), portosystemic collaterals-shunts at imaging (n = 9), ascites (n = 8), and esophageal varices (n = 6) (Fig. 3, Fig. 4A). These signs of portal hypertension were revealed by thrombocytopenia in five asymptomatic patients and by the development of ascites in the five others. Cumulative incidence of first signs of portal hypertension, considering non-liver-related death as a competing event, was 22%, 27%, and 44% at 1, 3, and 10 years, respectively (Fig. 4A). Details on the results of liver investigations, including the work-up for common causes of liver disease, and on hematological diseases and their treatment in these 10 patients who developed signs of portal hypertension, are presented in Tables S2 and S3. All four patients who had histological lesions of PSVD on biopsy diagnosed with SOS developed signs of portal hypertension during follow-up. Patients who did not develop signs of portal hypertension were followed for a median of 7.0 years (IQR, 3.4–8.6) after SOS.

Of the 10 patients who developed signs of portal hypertension, LSM was performed during follow-up in six patients (Patients #2, 10, 12, 15, 16, and 20), including two patients using 2D shear wave elastography and four using transient elastography. Among them, three had LSM value <8 kPa (Patients #12, 15, and 20) and three had LSM value ≥8 kPa (Patients #2, 10, and 16, having LSM values ranging from 11 to 18.6 kPa) (Table 2 and Table S4). Three of 10 patients who developed signs of portal hypertension (Patients #2, 12, and 16) underwent a second liver biopsy 4 years, 6 months, and 3 months after SOS diagnosis, respectively (Fig. 2 and Figs. S1 and S2). All biopsies (size ranging from 19 to 36 mm) showed the absence of advanced liver fibrosis or cirrhosis together with features consistent with PSVD, namely NRH in two patients (Patients #2 and 12), regenerative changes without clear NRH in one patient (Patient #16), and portal vein stenosis in ≥50% of the portal tract in all three patients. Therefore, all three patients met the diagnostic criteria for definite PSVD according to the 2026 PSVD diagnostic score.^10^ All three biopsies also showed mild-to-moderate periportal fibrosis, as well as perivenular and perisinusoidal fibrosis.

**Table 2:** Features of the patients with SOS during follow-up.

	Patients not developing signs of portal hypertension during follow-up (n = 12)		Patients developing signs of portal hypertension during follow-up (n = 10)		*p* values
	n =	n (%) or median (IQR)	n =	n (%) or median (IQR)	
**Laboratory data at the end of follow-up**					
Prothrombin time (%)	12	100 (95–100)	9	100 (48–100)	0.780
INR	12	1.01 (1.0–1.01)	9	1.11 (1.01–1.44)	0.621
Platelet count (x10^9^/L)	12	199 (155–286)	10	86 (48–129)	**0.008**
Serum AST (IU/L)	12	31 (11–44)	9	61 (38–119)	**0.030**
Serum ALT (IU/L)	12	33 (22–53)	9	54 (39–116)	0.670
Serum GGT (IU/L)	12	109 (36–246)	8	113 (65–284)	1.000
Serum total bilirubin (μmol/L)	10	7 (3–10)	10	15 (9–43)	**0.023**
Serum creatinine (μmol/L)	12	72 (55–102)	9	80 (55–153)	0.670
Serum albumin (g/L)	10	41 (37–43)	9	35 (25–45)	0.367

**Risk factors for liver disease present after HSCT**					
Metabolic risk factors at end FU∗ (%)	12	6 (50)	10	5 (50)	1.000
Alcohol use disorder (%)	12	1 (8)	10	3 (30)	0.281
BMI (kg/m^2^)	12	27 (25–28)	9	29 (24–30)	0.356

**Liver-related events during follow-up**					
Total follow-up duration (years)	12	7.0 (2.7–9.8)	10	3.8 (2.0–6.6)	0.111
Ascites (%)	12	0 (0)	10	8 (80)	**0.001**
Variceal bleeding (%)	12	0 (0)	10	3 (30)	**0.041**
Hepatic encephalopathy (%)	12	0 (0)	10	3 (30)	**0.041**
Spontaneous bacterial peritonitis (%)	12	0 (0)	10	1 (10)	0.262
Portal vein thrombosis (%)	12	0 (0)	10	1 (10)	0.262

**Features at the end of follow-up**					
Liver stiffness (kPa)	8	5 (4–6)	6	13 (6–15)	**0.017**
CAP value (dB/m)	6	190 (178–260)	6	310 (228–346)	**0.025**
Esophageal varices (%)	12	0 (0)	10	6 (60)	**0.001**
Large esophageal varices (%)	12	0 (0)	10	5 (50)	**0.001**

**Imaging findings**					
Pathological ultrasound (%)	12	0 (0)	10	10 (100)	**0.001**
Ultrasound features†: Abnormal liver morphology	12	0 (0)	10	8 (80)	**0.001**
Portosystemic collaterals	12	0 (0)	10	9 (90)	**0.001**
Ascites	12	0 (0)	10	5 (50)	**0.001**
Enlarged spleen size (≥13 cm in the largest axis)	12	0 (0)	10	4 (40)	**0.001**

For patients who died during follow-up, data presented here are the last available data before death. Data are presented as median (IQR) or n (%). Comparison of quantitative and qualitative variables was done using the Mann–Whitney *U* test, Χ^2^ test, or Fisher’s exact test, as appropriate. Bolded values indicate statistically significant differences (*p* <0.05).

ALT, alanine aminotransferase; AST, aspartate aminotransferase; FU, follow-up; GGT, gamma-glutamyl transferase; INR, international normalized ratio; SOS, sinusoidal obstructive syndrome.

∗ Metabolic risk factors: included overweight (BMI ≥25 kg/m^2^), diabetes mellitus, arterial hypertension, and/or dyslipidemia.

† Ultrasound features: abnormal liver morphology, ascites, portosystemic collaterals and enlarged spleen size (≥13 cm in the largest axis).

### Features associated with the development of signs of portal hypertension after SOS

Individual outcomes from the diagnosis of SOS to the end of follow-up in patients with SOS who developed or did not develop signs of portal hypertension are summarized in Table 2 and Fig. 3A,B and presented in detail in Table S4.

To identify characteristics associated with the development of signs of portal hypertension during the follow-up, we performed univariable and multivariable analysis, considering non-liver-related death as a competing event. As shown in Table S5, lack of treatment of SOS with defibrotide (subdistribution hazard ratio [sHR]: 0.096, 95% CI: 0.03–0.33) and lower mean arterial pressure at the time of SOS diagnosis (sHR: 0.95, 95% CI: 0.93–0.99), were independent predictors of development of signs of portal hypertension. As defibrotide obtained approval in Europe in 2013, we performed a sensitivity analysis restricted to patients included in 2013 and later (n = 14) and observed that absence of defibrotide treatment and lower mean arterial pressure remained independently associated with the development of signs of portal hypertension (data not shown).

Of note, during follow-up after HSCT, exposure to risk factors for chronic liver disease did not differ between the two groups (Table 2).

### Liver-related complications and long-term survival

Out of the 10 patients who developed signs of portal hypertension during follow-up in Fig. 4A, eight developed liver-related complications, including ascites (n = 8), hepatic encephalopathy (n = 3), and variceal bleeding (n = 3) (Fig. 3A). One patient developed portal vein thrombosis, and one developed spontaneous bacterial peritonitis. One patient (Patient #1) required a surgical portosystemic shunt as a result of variceal bleeding during the follow-up; no TIPS and no liver transplantation were performed. Median time between SOS diagnosis and first liver-related complications was 4.6 years (IQR 1.4–7.8). Cumulative incidence of liver-related complications, considering non-liver-related death as a competing event, was 0%, 21%, and 40% at 1, 3, and 10 years, respectively (Fig. 4A). None of the patients without signs of portal hypertension developed liver-related complications.

During the study period, seven patients (32%) died (six with portal hypertension and one without). Cumulative incidence of overall mortality was significantly higher in patients who developed signs of portal hypertension (10-year mortality of 71%) than in those without signs of portal hypertension (10-year mortality of 33%) (hazard ratio 10.7 [1.27–89.9]; *p* = 0.03] (Fig. 4B). Causes of death differed substantially between patients with and without portal hypertension during follow-up. In patients developing signs of portal hypertension, four deaths were liver-related (portal hypertension bleeding, hepatic encephalopathy) and two were as a result of progression of hematological disease. In patients not developing signs of portal hypertension, one death was reported and was associated with the persistence of hematological disease.

## Discussion

Our study shows that the development of portal hypertension and liver-related complications is common in the long term in patients with SOS related to HSCT, especially when patients did not receive defibrotide as a treatment of SOS. The development of signs of portal hypertension is associated with worse overall survival. Histologically, SOS may develop into PSVD.

The first major finding of this study was that patients with SOS have a high risk of developing signs of portal hypertension and liver-related complications in the long-term, namely 44% and 40% at 10 years after SOS, respectively. A previous study reported ascites in 40 out of the 4,660 (0.9%) patients after HSCT.^16^ However, the time interval between HSCT and ascites was short (median duration of 87 days), and the authors ruled out SOS in those patients. Other studies on portal hypertension after HSCT focused mainly on the short term after HSCT (typically 90 days) when multiple causes of ascites, including SOS, may be present^17^ or on cirrhosis that developed in 1–3% of the patients 10–20 years after HSCT, mainly because of HCV infection, without association with SOS.^18^ Our study shows that the appearance of signs of portal hypertension is a turning point in patients with a history of SOS after HSCT, as 10-year overall mortality after HSCT was 71% in patients who developed signs of portal hypertension *vs.* 33% in those who did not. This incidence rate is higher than that observed for patients with PSVD in general, in whom the cumulative incidence of death or liver transplantation is 28% at 10 years,^19^^,^^20^ and also higher than usually observed after HSCT.^21^ In practice, our data suggest that patients who survive after SOS in the context of HSCT should be followed in the long term for the development of signs of portal hypertension. Indeed, in cases of large gastroesophageal varices, beta-blocker prophylaxis for variceal bleeding could be considered, as recommended in patients with non-cirrhotic portal hypertension.^13^ We might propose in the 5 years after SOS: (i) liver blood tests and platelet count every 6 months; (ii) LSM, SSM, and abdominal imaging every year, even in the absence of blood test abnormalities; and (iii) upper gastrointestinal endoscopy in case of signs of portal hypertension, typically thrombocytopenia or splenomegaly.

The second major finding of this study is that SOS might, in the long term, evolve into PSVD. Indeed, three of our patients with SOS and portal hypertension had, after the initial liver biopsy showing SOS, a second liver biopsy with clear histological lesions of PSVD and no cirrhosis (Patients #2, 12, and 16) (Fig. 2 and Figs. S1 and S2). Out of the seven other patients who developed signs of portal hypertension, but who did not undergo liver biopsy, two (Patients #15 and 20) had specific signs of portal hypertension and LSM <10 kPa, so that PSVD was highly likely.^4^^,^^22^ Out of the five remaining patients, two (Patients #9 and 8) had signs of portal hypertension and no cause for cirrhosis, so that PSVD can also be strongly suspected; two patients (Patients #1 and 4) had signs portal hypertension, no LSM available, but excessive alcohol consumption, so that type of liver disease remains uncertain; the last patient (Patient #10) had an LSM value of 18.6 kPa, steatosis suspected at imaging and several risk factors for chronic liver disease (alcohol use disorder, overweight), so that cirrhosis is possible. Altogether, three patients had definite PSVD, four suspected PSVD, two uncertain diagnoses, and one probable cirrhosis. It should be noted that exposure to metabolic syndrome, known to be common after HSCT,^23^ was not different between patients developing or not having signs of portal hypertension. Outside the setting of HSCT, oxaliplatin, as a treatment of colorectal cancer, has been described to induce SOS in the short term, and PSVD in the long term, portal hypertension.^22^^,^^24^^,^^25^ The mechanisms by which SOS could lead to PSVD are unknown. We can, however, speculate that hepatic venous outflow obstruction induced by SOS could disrupt normal hepatic blood flow and lead to remodeling of the intrahepatic vasculature, as reported in Budd–Chiari syndrome^26^ and chronic cardiac failure.^27^ Importantly, four patients who developed signs of portal hypertension had histological lesions of PSVD at SOS diagnosis, suggesting that, in some patients, PSVD lesions may be present before SOS and may be attributable to the hematological disease or its treatment before HSCT. However, our study does not allow to determine the temporal relationship between these entities. Along these lines, Varma *et al.*^16^ reported histological lesions of PSVD in over half of 24 patients who underwent liver biopsies for unexplained ascites, without SOS, around 100 days after HSCT.

Overall, our findings suggest that the exclusion of survivors of SOS following HSCT from the PSVD diagnostic framework may warrant reconsideration, particularly beyond 6 months after HSCT.

The third major finding of this study was the identification of the absence of use of defibrotide to treat SOS, and of lower mean arterial pressure on the day of SOS diagnosis as independently predicting the development of signs of portal hypertension in the long-term after SOS. Studies available so far on defibrotide have focused on the first 3 months after HSCT, so data beyond day 100 were not available.^28^^,^^29^ Our study suggests a protective role of defibrotide treatment in patients with SOS following HSCT to prevent portal hypertension development in the long-term. Strikingly, nine out of the 10 patients who developed signs of portal hypertension were not treated with defibrotide. However, this observation should be interpreted with caution, and only as hypothesis-generating, because during such a long inclusion period (1994–2023), changes in selection criteria for HSCT, disease severity, and treatments are likely to have occurred. Further studies are needed to confirm or refute this finding. One possible reason for the association between lower mean arterial pressure and the long-term development of portal hypertension is that it reflects systemic circulatory dysfunction associated with more severe SOS. If this hypothesis were true, it would mean that SOS severity was not captured by histology (Table S1) nor EBMT (European Society for Blood and Marrow Transplantation) 2023 criteria (Table 1). Further studies are needed to confirm or refute this.

Finally, our study has some limitations: (i) the lack of a control group of patients who underwent HSCT, but did not develop SOS, to determine the incidence of signs of portal hypertension during follow-up in this population; (ii) the fact that only histologically proven SOS cases were included, so that these patients might not represent the full spectrum of SOS after HSCT; and (iii) the very small number of patients who had a second liver biopsy during follow-up. Moreover, the comparison between patients who developed or did not develop SOS during follow-up is also confounded by the very long inclusion period, during which time there may have been changes in diagnostic and therapeutic management. This study should therefore be considered as a proof of concept, exploratory study.

In conclusion, this study demonstrates that signs of portal hypertension and liver-related complications are common during long-term follow-up after SOS following HSCT. Treatment of SOS with defibrotide seems to be associated with a lower risk of developing portal hypertension. Liver histology at the long-term follow-up after SOS may show features consistent with PSVD. The development of portal hypertension and liver-related complications decreases long-term patient survival.

## Abbreviations

ALT, alanine aminotransferase; AST, aspartate aminotransferase; Bpm, beats per minute; BMI, body mass index; CAP, controlled attenuation parameter; EBMT, European Society for Blood and Marrow Transplantation; FU, follow-up; GGT, gamma-glutamyl transferase; HL, Hodgkin lymphoma; HSCT, hematopoietic stem cell transplantation; HVPG, hepatic venous pressure gradient; INR, international normalized ratio; IQR, interquartile range; K7, keratin 7; LSM, liver stiffness measurement; NHL, non-Hodgkin lymphoma; NRH, nodular regenerative hyperplasia; PH, portal hypertension; PSVD, porto-sinusoidal vascular disorder; SOS, sinusoidal obstruction syndrome; SSM, spleen stiffness measurement; VCTE, vibration-controlled transient elastography; VOD, hepatic veno-occlusive disease.

## Authors’ contributions

Study concept and design: EAT, PER. Acquisition of data: EAT, DM, AS, LB, VP, PER. Analysis and interpretation of data: AF, EAT, AS, VP, PER. Statistical analysis: AF, EAT, PER. Drafting the manuscript: EAT, PER, LB. Critical revision of the manuscript: all authors. Approved the final version of the article: all authors.

## Data availability

The authors are prepared to provide the data from this study upon request to the corresponding author.

## Financial support

P-ER’s research laboratory is supported by the Fondation pour la Recherche Médicale (FRM EQU202303016287), ‘Institut National de la Santé et de la Recherche Médicale’ (ATIP AVENIR), the ‘Agence Nationale pour la Recherche’ (ANR-18-CE14-0006-01, RHU QUID-NASH, ANR-18-IDEX-0001, and ANR-22-CE14-0002) by «Émergence, Ville de Paris», by Fondation ARC, by the European Union’s Horizon 2020 research and innovation program under grant agreement No 847949 (DECISION) and N°825575 (RiTa) and by France 2030 RHU LIVER-TRACK (ANR-23-RHUS-0014). EA-T is a recipient of a ‘Río Hortega’ fellowship grant from the Instituto de Salud Carlos III (CM16/00133), ‘Juan Rodés’ post-doctoral grant from the Instituto de Salud Carlos III (JR20/00047), the PI21/01995 grant from the ISCIII, and EURO-VALDI-NET COST Action CA-23146 Short Term Scientific Mission (STSM) Grant 2025.

## Conflicts of interest

P-ER has received research funding from Terrafirma and acted as a consultant for Mursla, Genfit, Boehringer Ingelheim, Cook, AstraZeneca, Jazz, and Abbelight, and received speaker fees from AbbVie. The remaining authors declare no conflicts of interest that pertain to this work.

Please refer to the accompanying ICMJE disclosure forms for further details.
