# Supplementary material for: Long-term outcome of sinusoidal obstruction syndrome secondary to hematopoietic stem cell transplantation
Source: JHEP Rep. 2026 Aug 6;8(10):101986. doi: 10.1016/j.jhepr.2026.101986 (PMC13598180; doi:10.1016/j.jhepr.2026.101986)

Long-term outcome of sinusoidal obstruction syndrome secondary to hematopoietic stem cell transplantation

Edilmar Alvarado-Tapias, Alexandre Sayadi, Lise Bertin, Aurélie Plessier, François Durand, Andreu Ferrero-Gregori, Valérie Paradis, David Michonneau, Pierre-Emmanuel Rautou

Table of contents

Table S1……………………………………………………………………………….2

Table S2……………………………………………………………………………….4

Table S3……………………………………………………………………………….5

Table S4……………………………………………………………………………….6

Table S5……………………………………………………………………………….9

Fig. S1………………………………………………………………………………..11

Fig. S2………………………………………………………………………………..13

Fig. S3………………………………………………………………………………..15

Table S1. Liver histological lesions at diagnosis of SOS.

	Overall	Portal hypertension during follow-up		
**Characteristic**	**N = 22**[1]	**No** N = 12[1]	**Yes** N = 10[1]	**p-value**[2]
SOS grade (%) (1/2/3)	5(23)/9(41)/8(36)	1(8)/5(42)/6(50)	4 (40)/4(40)/2(20)	0.2
Sinusoidal dilatation (%) (0/1/2/3)	3 (14)/7(32)/7(32)/5(23)	0(0)/4(33)/4(33)/4(33)	3(30)/3(30)/3(30)/1(10)	0.2
Perisinusoidal hemorrhage	16 (73)	10 (83)	6 (60)	0.3
Peliosis	6 (27)	5 (42)	1 (10)	0.2
Hepatocellular damage	7 (32)	6 (50)	1 (10)	0.074
Central vein lesion (%) (0/1/2)	10(45)/4(18)/8(36)	4(33)/4(33)/6(50)	6(60)/2(20)/2(20)	0.3
Central vein fibrosis (%) (0/1/2)	2(9)/13(59)/7(32)	2(17)/7(58)/3(25)	0(0)/6(60)/4(40)	0.6
Sinusoidal fibrosis (%) (0/1/2)	10(45)/9(41)/3(14)	8(67)/3(25)/1(8)	2(20)/6(60)/2(20)	0.087
Nodular regeneration (%) (0/1/2/3)	13(59)/5(23)/1(4)/3(14)	8(67)/4(33)/0(0)/0(0)	5(50)/1(10)/1(10)/3(30)	0.089
Steatosis (%) (0/1/2/3)	18(82)/3(14)/1(4.5)/0(0)	12(100)/0(0)/0(0)/0(0)	6(60)/3(30)/1(10)/0(0)	**0.029**
Steatohepatitis (%) (0/1)	22(100)/0(0)	12(100)/0(0)	10(100)/0(0)	
Cholestasis (%)	1 (4.5)	0 (0)	1 (10)	0.5
Portal vein lesion (0/1/2)	19(86)/3(14)/0(0)	10(83)/2(17)/0(0)	9(90)/1(10)/0(0)	0.9
Portal vein stenosis (%) (0/1/2)	13(59)/6(27)/3(14)	9(75)/3(25)/0(0)	4(40)/3(30)/3(30)	0.11
Portal vein fibrosis (0/1/2)	16(73)/3(14)/3(14)	11(92)/1(8)/0(0)	5(50)/2(20)/3(30)	0.091
Portal tract abnormalities	3 (14)	0 (0)	3 (30)	0.078
METAVIR Fibrosis (F0/F1/F2)	16(73)/4(18)/2(9)	12(100)/0(0)/0(0)	4(40)/4(40)/2(20)	**0.003**

Data are presented as median (interquartile range) or frequencies (proportion), as appropriate. Comparison of quantitative and qualitative variables was done using the Mann-Whitney or Fisher's exact tests, as appropriate. Bolded values indicate statistically significant differences (p<0.05). SOS, sinusoidal obstruction syndrome; NRH, nodular regenerative hyperplasia.

Definitions:

For sinusoidal dilatation, a semi-quantitative scoring defined a priori was used: 0, no lesion; 1, sinusoidal dilatation limited to centrilobular sinusoids; 2, extensive sinusoidal dilatation affecting centrilobular areas up to mediolobular sinusoids; 3, sinusoidal dilatation extending to periportal zones.

Perisinusoidal hemorrhage, peliosis, hepatocellular damage, and steatohepatitis were assessed as present or absent.

Nodular regeneration was assessed as follows: 0 (absent), 1 mild (focal occasionally distinct nodular hyperplasia on reticulin staining but indistinct on H&E staining); 2, moderate (focal distinct nodular hyperplasia apparent on H&E staining, clearly highlighted on reticulin staining); 3, severe (diffuse nodular hyperplasia, distinct in most areas on H&E staining and highlighted on reticulin staining, corresponding to nodular regenerative hyperplasia (NRH)).

For steatosis, a semi-quantitative assessment of the proportion of involved hepatocytes was used as follows: 0, absent; 1, mild (5-33%); 2, moderate (33-66%); and 3, severe (>66%).

Venular lesions and fibrosis were assessed as follows: 0, absent; 1, <50% of veins/portal tracts/sinusoids; and 2, ≥50% of veins/portal tracts/sinusoids throughout the whole biopsy.

METAVIR Fibrosis was assessed as follows: 0, absent; 1, portal fibrosis; 2, periportal and bridging fibrosis. No F3 (extensive bridging fibrosis) or F4 (cirrhosis) cases were seen.

Portal vein stenosis was assessed as follows: 0, absent; 1, <50% of portal veins; and 2, ≥50% of portal veins throughout the entire biopsy.

Portal tract abnormalities (i.e., hypervascularized portal tracts, herniated portal vein branches, and periportal abnormal vessels) were assessed as present or absent.

SOS grade was determined based on the extent of histological lesions associated with SOS: mild (centrolobular), moderate (centro- and mediolobular), or severe (panlobular).

Table S2. Detailed liver blood tests, imaging features and risk factors for liver diseases before conditioning for HSCT for the 10 patients who developed signs of portal hypertension during follow-up.

Patient	Platelet count ($x10^9/L$)	INR	Serum AST (IU/L)	Serum ALT (IU/L)	Serum GGT (IU/L)	Serum bilirubin (µmol/L)	Serum creatinine (µmol/L)	Serum albumin (g/L)	[a]US/CT scan	Toxic-metabolic Risk factors	[b] HBs Ag	[b] Anti-HCV Ab	[b] Anti-HIV Ab
2	89	1.3	47	43	123	10	56	NA	Normal	DM, steatosis, obesity	No	No	No
15	185	1.0	42	45	39	7	56	41	Normal	No	No	No	No
10	240	1.0	17	22	192	4	73	40	No lesion	AUD+ steatosis + overweight	No	No	No
9	169	1.0	36	35	74	6	50	38	Liver lymphoma	No	No	No	No
12	91	1.0	49	56	109	14	77	30	Liver lymphoma	Obesity	No	No	No
1	45	1.20	42	36	37	5	89	37	Normal	AUD, overweight	No	No	No
16	89	1.0	32	32	70	5.7	65	NA	Normal	DM, obesity	No	No	No
20	64	1.0	52	55	32	8.5	36	41	Normal	No	No	No	No
4	125	1.4	67	62	45	11	58	29	Normal	AUD	No	No	No
8	80	1.1	57	47	67	14	56	32	Normal	No	No	No	No

Data are presented as numbers.

Definitions.

[a] ***US-CT scan:*** *liver ultrasound, or abdominal CT scan*

[b] ***Pre-HSCT viral study: Hepatitis B virus (HBV):*** *Hepatitis B surface antigen (HBsAg), antibodies against surface antigen (anti-HBs), antibodies against core antigen (anti-HBc);* ***Hepatitis C virus (HCV):*** *antibodies (IgM-IgG) against hepatitis C virus (anti-HCV);* ***Human Immunodeficiency Virus (HIV):*** *antibodies against HIV 1 and 2 and p24 antigen test.*

Abbreviations: AUD: *alcohol use disorder;* ***AST:*** *aspartate aminotransferase level;* ***ALT:*** *alanine aminotransferase level;* ***DM:*** *diabetes mellitus;* ***GGT:*** *gamma-glutamyl transferase;* ***INR:*** *international normalized ratio;* ***US:*** *liver ultrasound;* ***CT scan:*** *abdominal computed tomography.*

Table S3. Hematological disease and conditioning before HSCT in the patients, 10 developed signs of portal hypertension during follow-up.

Patient	Hematological disease	[a] Conditioning	Evolution of hematological disease	[b]Evolution
2	Acute myeloid leukemia	Bu (1 AutoHSCT)	Complete remission	30 years after HSCT, alive without clinical complication
15	Myelodysplastic syndrome	CsA (1 AlloHSCT)	Complete remission	7 years after HSCT, alive without complication
10	Chronic lymphatic leukemia	Bendamustine	Chronic lymphatic leukemia resistant to bendamustine, with a second line of ibrutinib and rituximab.	7 years after HSCT conditioning, death due to chronic hematological disease.
9	Hodgkin lymphoma (liver and spleen affectation)	Bu (1 AutoHSCT)	Complete remission	6 years after HSCT, death due to liver-related complications
12	Diffuse Large B-Cell Lymphoma (liver involvement)	Cy +Dacarbazine	Complete remission	5 years after HSCT conditioning, alive, with liver-related complications.
1	Acute myeloid leukemia	Bu (1 AutoHSCT)	Complete remission	4 years after HSCT, death due to liver-related complications
16	Acute myeloid leukemia	Bu (1 AlloHSCT)	Graft versus host disease	30 months after HSCT, death due to graft versus host complications, bronchiolitis with pneumothorax and neurological affectation
20	Acute lymphoid leukemia	Inotuzumab+ CsA (1 AlloHSCT)	Complete remission	2 years after HSCT, alive, with liver-related complications.
4	Diffuse Large B-Cell Lymphoma	Bu (2 AutoHSCT)	Complete remission	21 months after HSCT, death due to liver-related complications.
8	Diffuse Large B-Cell Lymphoma	Bu (2 AutoHSCT)	Complete remission	18 months after HSCT, death due to liver-related complications

[a] ***Conditioning:*** *included the pharmacological treatment used before HSCT. Bu*, busulfan; CsA: cyclosporine; Cy, cyclophosphamide.

[b] ***Evolution:*** *included total follow-up and the clinical evolution with or without liver-related complications: ascites, variceal bleeding, hepatic encephalopathy, portal vein thrombosis, and spontaneous bacterial peritonitis.*

Abbreviations: AlloHSCT: *allo hematopoietic stem cell transplantation;* ***AutoHSCT:*** *auto hematopoietic stem cell transplantation;* ***HSCT:*** *hematopoietic stem cell transplantation;* ***FU****: follow-up.*

Table S4. Detailed features of the patients with SOS at the end of follow-up.

Patient	Signs of portal hypertension (date of appearance)	Stiffness measurement	CAP (dB/m)	US-CT scan during follow-up.	Gastric or esophageal varices	Liver-related complications	Vital status and duration of FU.<br>End of FU: Months.
**PATIENTS DEVELOPING SIGNS OF PORTAL HYPERTENSION DURING FOLLOW-UP (N=10)**							
2	Yes,<br>M: 240	M: 240<br>LSM 2D-SWE: 13 kPa<br>SSM: NA	NA	M: 359.<br>Steatosis and porto-systemic collaterals	No endoscopy performed.	No	M:360.<br>Alive without decompensation.
15	Yes,<br>M: 47	M:46<br>LSM TE: 6.6 kPa<br>SSM: 34 kPa	M:85<br>226	M: 83.<br>Dysmorphic liver (left lobe hypertrophy; peripheral atrophy of the right lobe)<br>Irregular echostructure and “blunt surface”<br>Splenomegaly (13 cm)<br>Porto-systemic collaterals	No endoscopy performed.	No	M:84<br>Alive
10	Yes,<br>M: 45.	M:47.<br>LSM TE: 18.6 kPa<br>SSM: 95 kPa	M:47<br>345	M: 76.<br>Steatosis<br>Splenomegaly (16.5 cm)<br>Porto-systemic collaterals<br>Ascites.	M:77.<br>Large esophageal varices.	M:68. Ascites.	M:80<br>Death due to relapsed hematological disease.
9	Yes,<br>M: 48	NA	NA	M: 81.<br>Dysmorphic liver (right lobe atrophy + segment I hypertrophy)<br>Ascites<br>Porto-systemic collaterals.	M:47.<br>Small varices.	M: 46. Ascites.<br>M: 82. SBP.<br>M:82. HE.	M:82<br>Death due to SBP+ HE.
12	Yes,<br>M: 9	M:59.<br>LSM TE: 7.2 kPa<br>SSM:78.7kPa	M:46<br>249	M: 60.<br>Dysmorphic liver (peripheral atrophy + central hypertrophy)<br>Porto-systemic collaterals<br>Splenomegaly (17 cm)<br>Low to moderate ascites.<br>Portal vein thrombosis.	M: 21<br>Large esophageal varices.	M: 15. Ascites.<br>M: 46. Portal vein thrombosis treated with rivaroxaban	M: 61<br>Alive.
1	Yes,<br>M:12	NA<br>NA	NA	M: 14.<br>Dysmorphic liver (right lobe atrophy + segment I hypertrophy)<br>surgical shunt. Scant ascites.<br>Porto-systemic collaterals	M:13<br>Large esophageal varices	M: 14. Ascites.<br>M: 14. Digestive bleeding.<br>M: 15. Surgical shunt.	M:30<br>Death due to digestive bleeding, and HE.
16	Yes,<br>M: 9	M:9<br>LSM 2D SWE: 12 kPa<br>LSM TE: 11 kPa<br>SSM: NA	M:10<br>350	M: 27.<br>Dysmorphic liver (left lobe hypertrophy; peripheral atrophy of the right lobe)<br>Irregular echostructure and “blunt surface”	No endoscopy performed.	No	M:30<br>Death due to leukemia complications, pulmonary and neurological involvement.
20	Yes,<br>M: 17	M:17<br>LSM TE: 5.1 kPa	M:17<br>324	M: 18.	No varices on the endoscopy.	M:18. Ascites.	M:21<br>Alive

		NA		Dysmorphic liver (right lobe atrophy + segment I hypertrophy)<br>Porto-systemic collaterals. Moderate ascites.			
4	Yes,<br>M: 12	NA<br>NA	NA	M: 13.<br>Dysmorphic liver (right liver atrophy + segment I hypertrophy). Porto-systemic collaterals	M: 15<br>Large esophageal varices	M:15. Ascites.<br>M: 23 Variceal bleeding, HE.	M:24.<br>Death due to variceal bleeding.
8	Yes,<br>M:8	NA<br>NA	NA	M:9<br>Dysmorphic liver (peripheral atrophy + central hypertrophy)<br>Ascites<br>Splenomegaly (13cm)<br>Porto-systemic collaterals	M: 11<br>Large esophageal varices	M: 12. Ascites.<br>M: 23. Variceal bleeding.	M:24.<br>Death due to variceal bleeding.
**PATIENTS NOT DEVELOPING SIGNS OF PORTAL HYPERTENSION DURING FOLLOW-UP (N= 12)**							
3	No signs of portal hypertension	M:340<br>LSM TE: 5.3 kPa<br>22 kPa	M:340<br>185	M: 341<br>Normal liver	No endoscopy performed.	No	M:352<br>Alive
21	No signs of portal hypertension	M:48<br>LSM 2D-SWE: 5.4 kPa<br>NA	NA	M:52.<br>Normal liver	No endoscopy performed.	No	M:132<br>Alive
13	No signs of portal hypertension	NA<br>NA	NA	M:55<br>Normal liver	No endoscopy performed.	No	M:135<br>Death due to the persistent hematological disease.
5	No signs of portal hypertension	NA<br>NA	NA	M:47<br>Normal liver	No endoscopy performed.	No	M:101<br>Alive
6	No signs of portal hypertension	M:21<br>LSM TE: 4.2 kPa<br>NA	NA	M:21<br>Normal liver	No endoscopy performed.	No	M:95<br>Alive
11	No signs of portal hypertension	M:27<br>LSM TE 6 kPa<br>NA	M:27<br>180	M:27<br>Normal liver	No endoscopy performed.	No	M:97<br>Alive
14	No signs of portal hypertension	NA<br>NA	NA	M:45<br>Normal liver	No endoscopy performed.	No	M:81<br>Alive
18	No signs of portal hypertension	M:22<br>LSM TE: 5.2 kPa<br>NA	M:22<br>195	M:22<br>Normal liver	No endoscopy performed.	No	M:28<br>Alive
17	No signs of portal hypertension	M:45<br>LSM TE: 4.9 kPa<br>NA	M:45<br>275	M:45<br>Normal liver	No endoscopy performed.	No	M:52<br>Alive
7	No signs of portal hypertension	NA<br>NA	NA	M:16<br>Normal liver	No endoscopy performed.	No	M:36<br>Alive

22	No signs of portal hypertension	M:12<br>LSM TE: 3.3 kPa<br>NA	M:13<br>170	M:13<br>Normal liver	No endoscopy performed.	No	M:13<br>Alive
23	No signs of portal hypertension	M:14<br>LSM TE: 5.6 kPa<br>NA	M:15<br>255	M:15<br>Normal liver	No endoscopy performed.	No	M:15<br>Alive

Data are presented as numbers. The liver and spleen stiffness were measured by transient elastography (TE) or 2D shear wave elastography (2D-SWE).

Abbreviations: 2D-SWE: 2D shear wave elastography, CAP: controlled attenuation parameter, HE: hepatic encephalopathy, LSM: liver stiffness measurement, SSM: spleen stiffness measurement, SBP: spontaneous bacterial peritonitis, TE: transient elastography.

For each feature, “M” represents the time interval in months since diagnosis of SOS.

Table S5. Univariate and multivariate competing-risks regression analyses to determine predictors of portal hypertension signs after HSCT during follow-up.

**Characteristics**	**Univariate analysis sHR (95%CI)**	***P* value**	**Multivariate analysis sHR (95%CI)**	***P* value**
**Age (years)**	1.015 (0.969-1.063)	0.530		
**Male sex (%)**	0.943 (0.244-3.641)	0.930		
**[a] Metabolic risk factors (%)**	1.275 (0.401-4.054)	0.680		
**History of Alcohol use disorder (%)**	**3.851 (1.095-13.541)**	**0.036**		
**Body mass index (kg/m^2)**	1.052 (0.939-1.178)	0.390		
**[c]Allogenic HSCT/AutoHSCT/no(%)**	0.841 (0.212-3.332)	0.810		
**Time between conditioning or HSCT and signs of SOS (days)**	1.001 (0.986-1.017)	0.850		
**Time between conditioning or HSCT and liver biopsy showing SOS (days)**	1.001 (0.993-1.009)	0.770		
**Defibrotide (%)**	**0.139 (0.022-0.876)**	**0.036**	**0.096 (0.028-0.329)**	**<0.001**
**Ursodeoxycholic acid (%)**	0.358 (0.095-1.351)	0.130		
**Liver stiffness baseline (kPa)**	0.989 (0.955-1.023)	0.510		
**Spleen size (larger axis) (cm)**	1.056 (0.786-1.418)	0.720		
**Features at SOS diagnosis by liver biops**				
Total bilirubin≥ 2mg/dl (%)	0.644 (0.188-2.203)	0.480		
**Weight gain>5% (%)**	2.554 (0.709-9.198)	0.150		
**Recent bacterial infection (%)**	0.741 (0.202-2.716)	0.650		
**Ascites (%)**	2.744 (0.802-9.388)	0.110		
**Laboratory data on the day of liver biopsy**				

Prothrombin time (%)	1.026	(0.992-1.061)	0.140		
INR	0.232	(0.008-6.622)	0.390		
Platelet count (x10⁹/L)	1.001	(0.994-1.008)	0.830		
Serum AST (IU/L)	0.993	(0.982-1.005)	0.240		
Serum ALT (IU/L)	0.993	(0.982-1.005)	0.250		
Serum GGT (IU/L)	0.998	(0.994-1.003)	0.470		
Serum total bilirubin (µmol/L)	1.003	(0.997-1.009)	0.340		
Serum creatinine (µmol/L)	1.005	(0.985-1.025)	0.640		
Serum albumin (g/L)	1.004	(0.805-1.253)	0.970		
**HVPG (mmHg)**	0.984	(0.915-1.058)	0.660		
**Right Atrial Pressure (mmHg)**	1.069	(0.893-1.279)	0.470		
**Heart rate (bpm)**	1.009	(0.984-1.034)	0.480		
**Mean arterial pressure (mmHg)**	**0.964**	**(0.939-0.989)**	**0.005**	**0.954 (0.925-0.985)**	**0.004**

The univariate and multivariate analyses were performed using competing risks regression to determine predictors of portal-hypertension signs, with non-liver-related events as competing risks. Data are presented as Sub-Hazard ratio (sHR) and CI 95% (confidence interval).

[a]Metabolic risk factors included overweight (BMI ≥25 kg/m²), diabetes mellitus, arterial hypertension, and/or dyslipidemia.

[b] HL: Hodgkin Lymphoma; NHL, non-Hodgkin Lymphoma

[c]Allo HSCT: allogenic hematopoietic stem cell transplantation; AutoHSCT: auto hematopoietic stem cell transplantation.

***Abbreviations:** AST: aspartate aminotransferase level; ALT: alanine aminotransferase level; GGT: gamma-glutamyl transferase; HSCT: hematopoietic stem cell transplantation; HVPG: hepatic venous pressure gradient; SOS: sinusoidal obstruction syndrome.*

LIVER BIOPSY AT SOS DIAGNOSIS

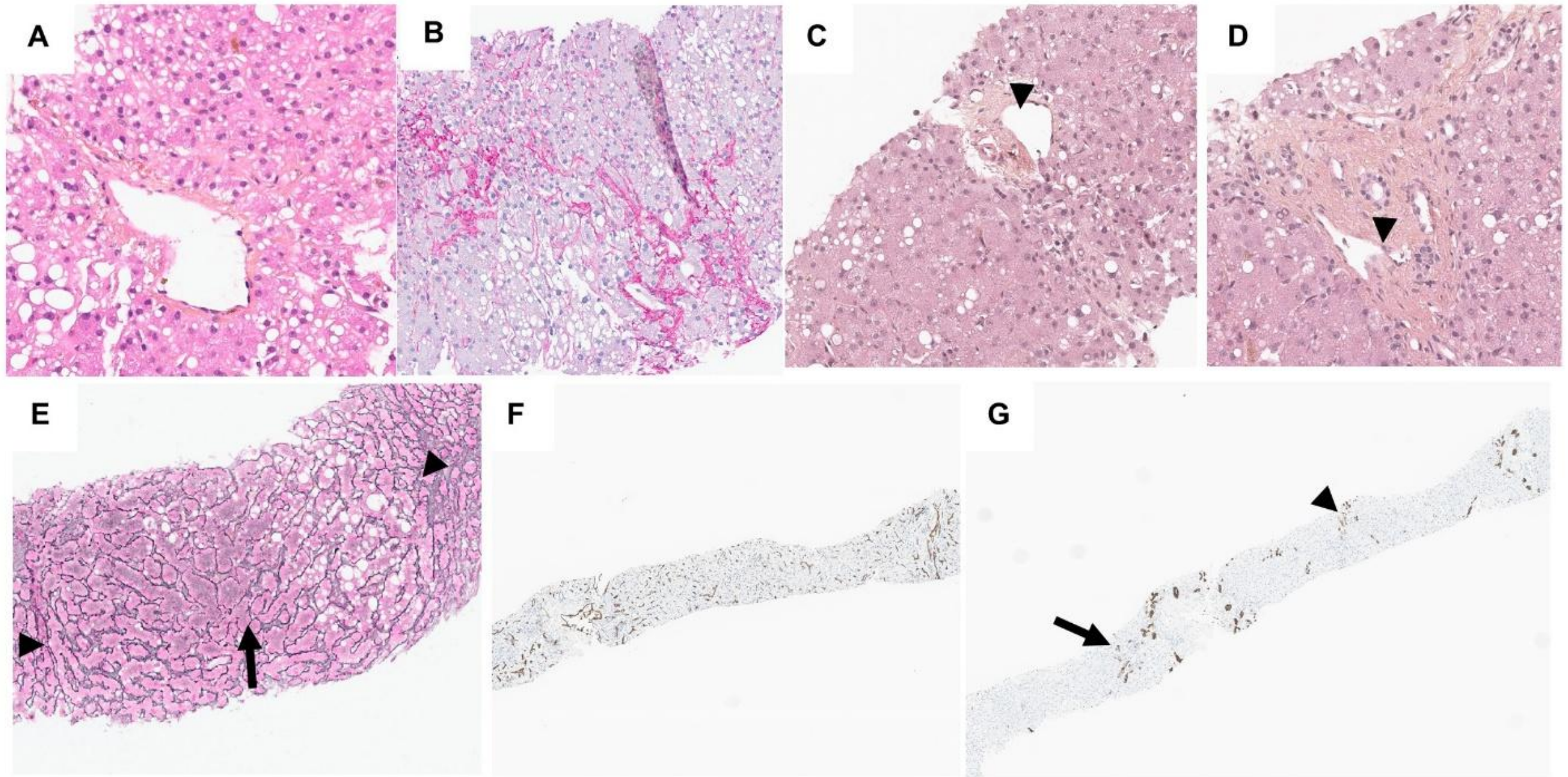

SECOND LIVER BIOPSY (6 MONTHS LATER) : DIAGNOSIS OF PSVD

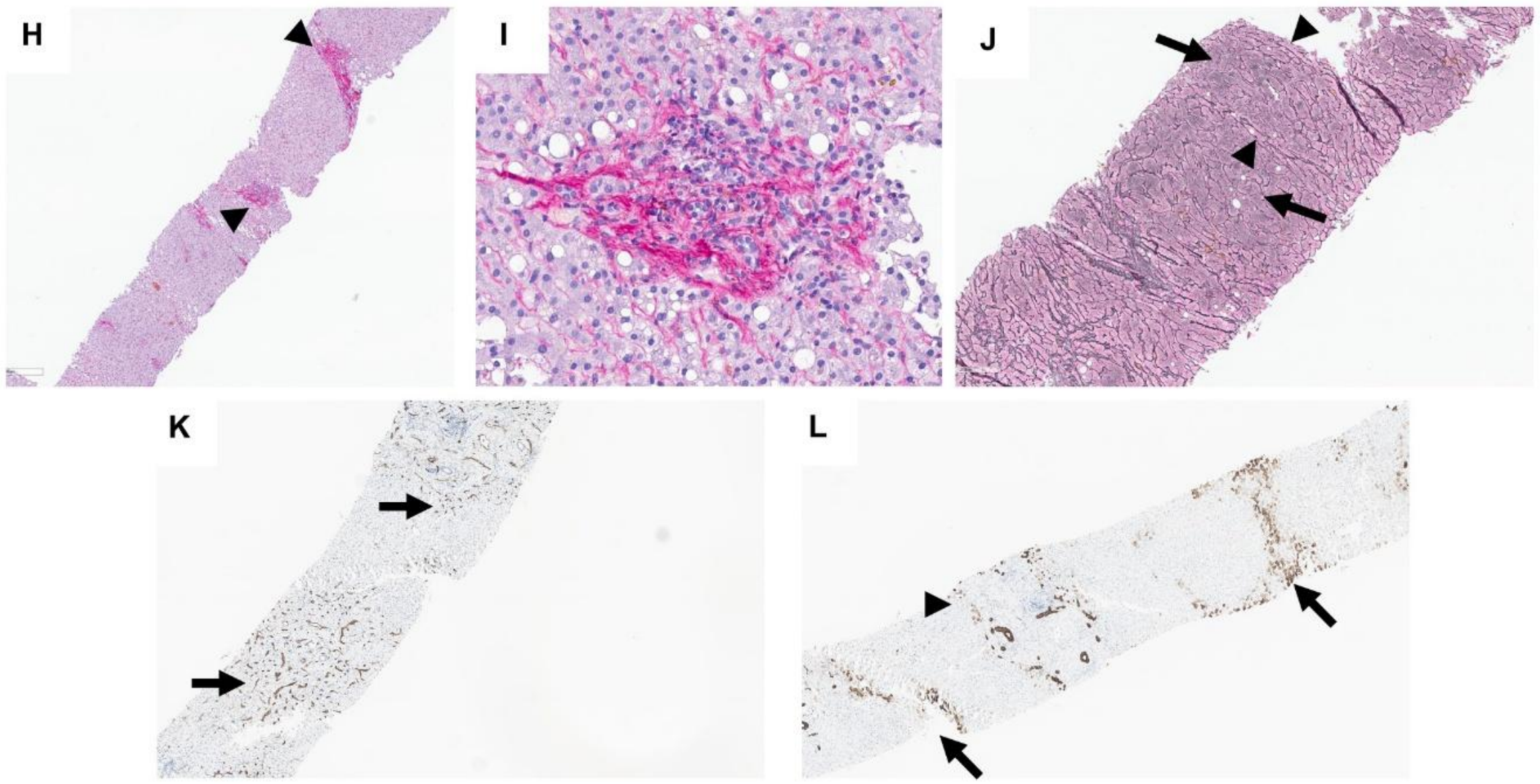

Fig. S1 legend: Patient #12. A. Hematein-Eosin-Safran (HES) stain, x200; **B.** Picro Sirius Hematoxylin (PSH), x100. This patient had SOS with chronic features on histology: few or no sinusoidal dilatations and prominent perivenular and perisinusoidal fibrosis. **C.** HES stain, x150. Normal portal tract with normal portal venule (arrowhead). **D.** HES stain, x150. Portal vein stenosis was seen in over 50% of portal tracts (arrowhead). **E.** Reticulin stain, x100. NRH features with foci of regenerative hepatocyte plates (arrows) surrounded by atrophic ones (arrowheads). **F.** CD34 immunostaining, x50. Diffuse sinusoidal capillarization. **G.** Keratin 7 (K7) immunostaining, x50. K7 highlights a few ischemic hepatocytes in zone 1 (arrow) and zone 3 (arrowhead). **H.** PSH stain, x20. Low magnification shows periportal fibrosis (arrowhead) without septal fibrosis or cirrhosis. **I.** PSH stain, x200. In the majority of portal tracts, portal veins were not seen. **J.** Reticulin stain, x50. Persistence of NRH features with foci of regenerative hepatocyte plates (arrow) surrounded by atrophic ones (arrowheads). **K.** CD34 immunostaining, x50. CD34 highlights zone 1 and 2 capillarization (arrows). **L.** K7 immunostaining, x50. Ischemic hepatocytes are mainly localized in zone 3 (arrows), with a few in zone 1 (arrowhead).

LIVER BIOPSY AT SOS DIAGNOSIS

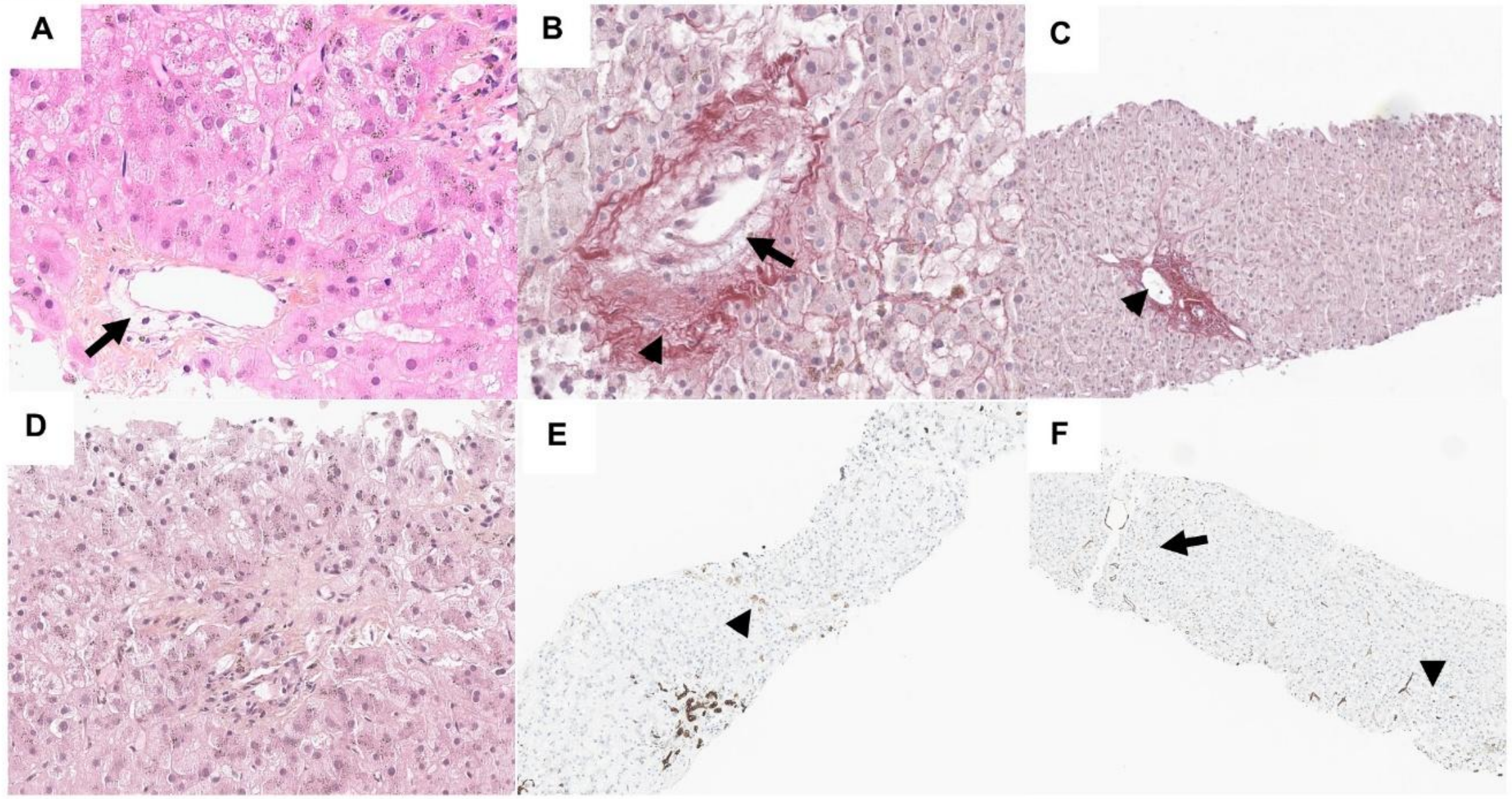

SECOND LIVER BIOPSY (3 MONTHS LATER) : DIAGNOSIS OF PSVD

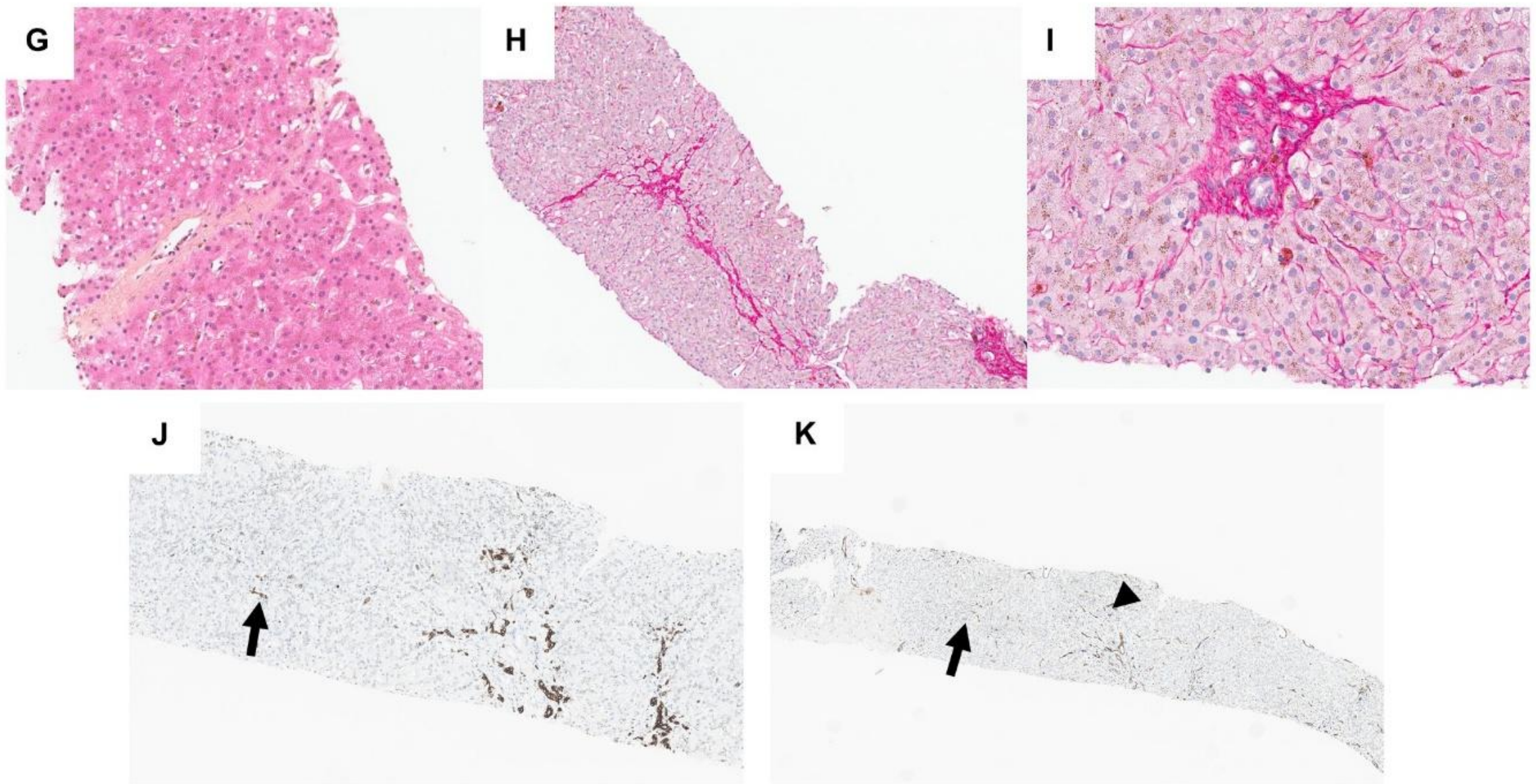

Fig. S2 legend: Patient #16.
Baseline biopsy:
A. Hematein-Eosin-Safran (HES) staining, x200; **B.** Picro Sirius Hematoxylin (PSH) staining, x200. SOS lesions were limited to centrolobular veins with fibrous thickening (arrowheads) and edema (arrows) of central veins, without sinusoidal dilatation. **C.** PSH staining, x100. **D:** HES staining, x200. The majority of portal tracts had easily identifiable portal veins (C), while a minority of portal tracts lacked a portal vein (D). **E.** Keratin 7 (K7) immunostaining, x100. K7 reveals a few zone 3 ischemic hepatocytes (arrowhead). **F.** CD34 immunostaining, x100. CD34 highlights sinusoidal capillarization in zone 1 (arrowhead) and zone 3 (arrow).
Second liver biopsy:
G. HES stain, x100; **H.** PSH stain, x50. Fibrous thickening of central veins was associated with perisinusoidal fibrosis and focal septal fibrosis, without cirrhosis. **I.** Picro Sirius Hematoxylin stain, x200.The majority of portal tracts lacked a portal vein (vanished portal veins), suggesting PSVD. **J**. (K7 immunostaining, x50) and **K** (CD34 immunostaining, x50) showed the same profile of expression as the baseline biopsy.

Fig. S3.

A) Cumulative incidence of mortality due to non-liver-related events by competing risk analysis in SOS patients during follow-up.

B) Cumulative incidence of mortality due to liver-related events by competing risk analysis in SOS patients during follow-up.

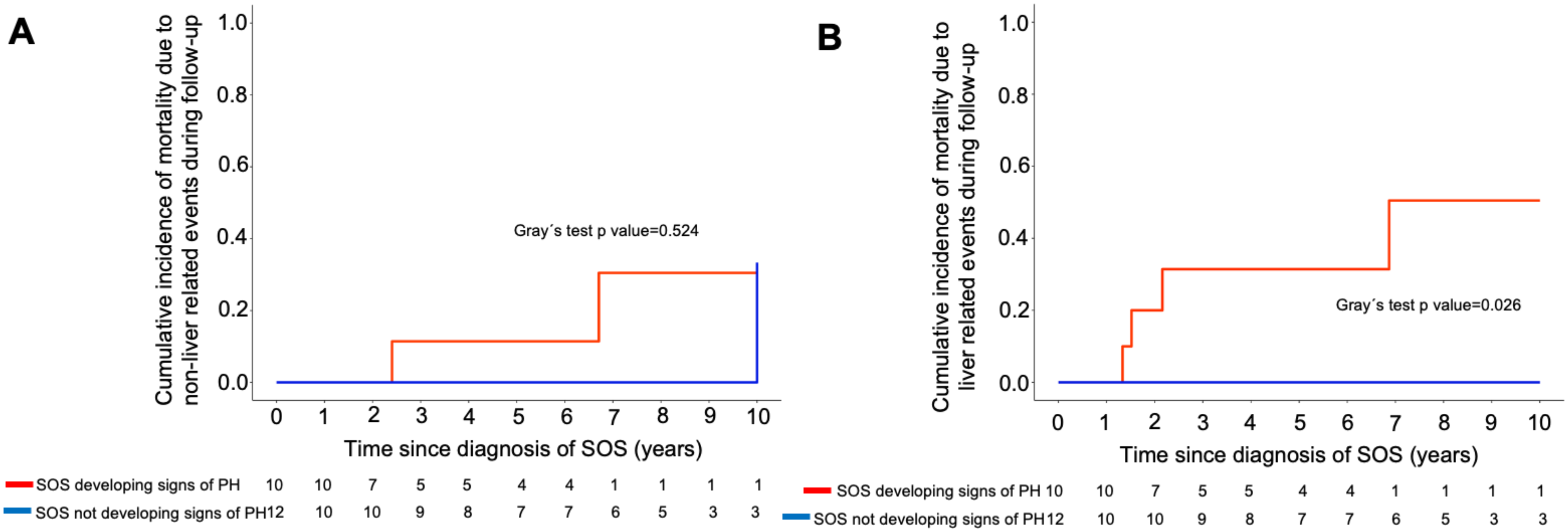

Supplement: Supplementary file 1 [file mmc1.pdf]
